# Molecular Functionalization of 2H-Phase MoS_2_ Nanosheets via an Electrolytic Route for Enhanced Catalytic Performance

**DOI:** 10.1021/acsami.1c08850

**Published:** 2021-07-12

**Authors:** S. García-Dalí, J. I. Paredes, S. Villar-Rodil, A. Martínez-Jódar, A. Martínez-Alonso, J. M. D. Tascón

**Affiliations:** Instituto de Ciencia y Tecnología del Carbono, INCAR-CSIC, Francisco Pintado Fe 26, 33011 Oviedo, Spain

**Keywords:** two-dimensional (2D) material, transition metal dichalcogenides (TMDs), MoS_2_, electrochemical exfoliation, colloidal dispersion, functionalization, catalytic reduction

## Abstract

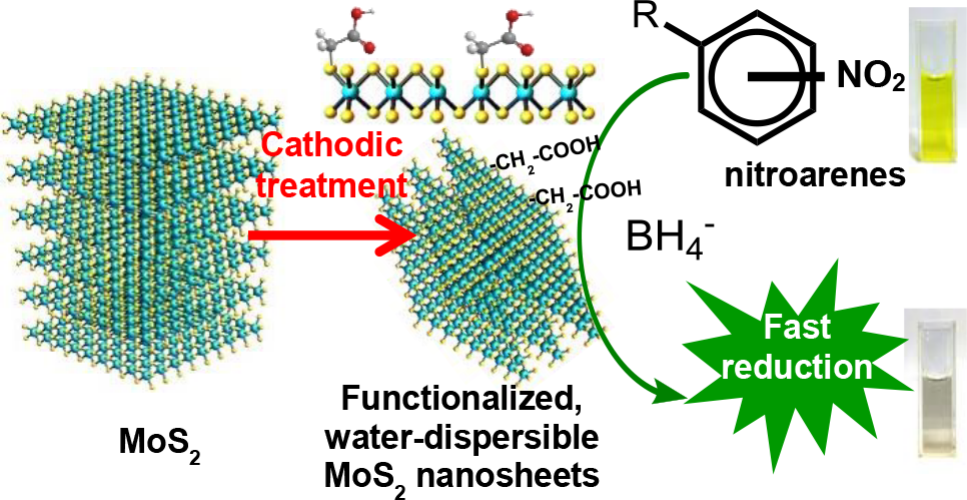

The molecular functionalization of two-dimensional MoS_2_ is of practical relevance with a view to, for example, facilitating its liquid-phase processing or enhancing its performance in target applications. While derivatization of metallic 1T-phase MoS_2_ nanosheets has been relatively well studied, progress involving their thermodynamically stable, 2H-phase counterpart has been more limited due to the lower chemical reactivity of the latter. Here, we report a simple electrolytic strategy to functionalize 2H-phase MoS_2_ nanosheets with molecular groups derived from organoiodides. Upon cathodic treatment of a pre-expanded MoS_2_ crystal in an electrolyte containing the organoiodide, water-dispersible nanosheets derivatized with acetic acid or aniline moieties (∼0.10 molecular groups inserted per surface sulfur atom) were obtained. Analysis of the functionalization process indicated it to be enabled by the external supply of electrons from the cathodic potential, although they could also be sourced from a proper reducing agent, as well as by the presence of intrinsic defects in the 2H-phase MoS_2_ lattice (e.g., sulfur vacancies), where the molecular groups can bind. The acetic acid-functionalized nanosheets were tested as a non-noble metal-based catalyst for nitroarene and organic dye reduction, which is of practical utility in environmental remediation and chemical synthesis, and exhibited a markedly enhanced activity, surpassing that of other (1T- or 2H-phase) MoS_2_ materials and most non-noble metal catalysts previously reported for this application. The reduction kinetics (reaction order) was seen to correlate with the net electric charge of the nitroarene/dye molecules, which was ascribed to the distinct abilities of the latter to diffuse to the catalyst surface. The functionalized MoS_2_ catalyst also worked efficiently at realistic (i.e., high) reactant concentrations, as well as with binary and ternary mixtures of the reactants, and could be immobilized on a polymeric scaffold to expedite its manipulation and reuse.

## 1 Introduction

Over the past decade, layered transition metal dichalcogenides (TMDs) in the form of nanosheets (NSs) have become one of the most intensively investigated members of the family of two-dimensional (2D) materials. Such a strong interest in 2D TMDs is rooted in their unique and wide-ranging physical properties as well as in their considerable promise for impactful applications in many key technological areas, including (opto)electronics, (photo-/electro)catalysis, electrochemical energy storage, chemical sensing, and biomedicine.^1^ While these 2D compounds can be useful for many practical purposes already in their pristine, unmodified configuration, it is generally accepted that reaching their full potential will require resorting to chemically modified variants, which can be accessed through heteroatom doping^2^ or molecular functionalization.^3,4^ For instance, substitutional doping of TMD NSs with selected heteroatoms (mainly other transition metal and chalcogen atoms, but also phosphorus, nitrogen, or chlorine) allows fine-tuning their electronic structure and, as a result, can lead to enhanced performance when they are used as components of electronic devices or as electrocatalysts for industrially relevant reactions (e.g., hydrogen evolution).^2^ Likewise, functionalization with proper molecular groups is an effective means to engineer the interaction of 2D TMDs with their surrounding environment, which facilitates their colloidal dispersion and processing in the liquid phase, their detection of analytes with high sensitivity and selectivity in sensing applications, or their uptake by cells when used as a carrier for drug delivery, to name a few examples.^4^

The molecular functionalization of 2D TMD NSs can be carried out by either physisorption (noncovalent) or chemisorption (covalent) strategies.^4,5^ The former are particularly widespread as a tool to improve their dispersibility in solvents or to modulate their charge carrier density, and they rely on van der Waals and/or charge transfer interactions to incorporate surfactants, polymers, and other (bio)molecules on the TMD surface. Chemisorption approaches, on the other hand, are comparatively much less prevalent. At least for the most commonly explored 2D TMDs, that is, mainly MoS_2_ but also WS_2_ or MoSe_2_, this fact can be attributed to the general lack of dangling bonds on their pristine basal surface and to the semiconducting nature of their thermodynamically stable 2H phase. Such features imply that these 2H-phase TMDs are rather chemically inert and thus not especially prone to take part in bond-forming processes.^3^ To circumvent this limitation, researchers have turned to the 1T (or 1T′)-phase counterparts of 2D MoS_2_, WS_2_, or MoSe_2_. The latter are metallic in nature (i.e., more electron-rich) and tend to possess substantial amounts of structural defects and imperfections (chalcogen vacancies, cracks, pinholes) as a result of their preparation mode (typically, lithium intercalation/exfoliation routes), making them considerably more reactive than their 2H-phase equivalents.^4,6,7^ As a matter of fact, these metallic 1T-phase TMDs have been successfully functionalized via reaction with organothiols,^6^ organoiodides,^8,9^ and aryl diazonium salts.^10^

Although metallic 1T-phase TMD NSs and their chemically functionalized derivatives are relevant materials on their own merit,^11,12^ they suffer from a number of drawbacks when compared to their 2H-phase versions. These include structural instability (the 1T phase is metastable), higher propensity to environmental oxidation and degradation, or more stringent preparation conditions (e.g., need to work under inert atmosphere during the lithium intercalation step).^11^ By heat treatment at moderate temperatures (∼150–300 °C), the functionalized 1T-phase NSs can in some cases be converted back to the 2H phase while retaining at the same time many of their grafted molecular groups.^8,9^ Still, the multistep nature of the overall process makes it unattractive with a view to its practical implementation. Hence, several research efforts have been made in recent years to explore the direct covalent functionalization of semiconducting 2H-phase TMDs, especially MoS_2_. As a result, the derivatization of 2H-phase MoS_2_ NSs using organothiols or dithiolanes,^13,14^ aryl diazonium salts,^15^ metal complexes,^16^ and maleimides^17^ has been shown to be feasible, mainly by drawing on common organic chemistry protocols carried out in nonaqueous solvents (alcohols, acetonitrile). It is noteworthy, however, that despite their potential intrinsic advantages, except for a very recent report,^18^ electrochemical or electrolytic methods for the functionalization of 2D TMDs (either 2H- or 1T-phase) have not yet been documented. Electrochemical strategies for molecular functionalization, and more generally for organic synthesis, are often more attractive than their classical, reagent-based counterparts in terms of, for example, atom economy and environmental friendliness (chemical redox agents being replaced by electrons) or industrial scalability, particularly if carried out in water.^19^ Thus, the availability of electrolytic routes toward chemically derivatized 2D TMDs could ease the deployment of such materials in practical uses.

Here, we address this gap and report the electrolytic functionalization of 2H-phase MoS_2_ NSs based on their reaction with organoiodides. While metallic 1T-phase MoS_2_ has been previously derivatized with this type of reagents via direct reaction, the latter was thought to be made possible by the presence of excess electrons in the NSs derived from the lithium intercalation step as well as by the relatively high chemical reactivity associated with the metallic phase.^7–9^ None of these features can be found a priori in semiconducting 2H-phase MoS_2_. However, we show that with an external supply of electrons (typically in the form of an electrical current, although proper reducing agents can also be used), the functionalization of semiconducting MoS_2_ NSs with organoiodides is possible. Importantly, we also demonstrate this derivatization route to be highly beneficial for the 2H-phase MoS_2_ NSs application-wise. Specifically, the functionalized NSs exhibited a much enhanced performance when used as a catalyst for the reduction of nitroarenes and organic dyes, with catalytic activity values that surpassed not only those of most previously reported MoS_2_-based catalysts but also those of most non-noble metal-based catalysts in general. Thus, the present work illustrates both the feasibility of functionalizing 2H-phase TMD NSs by electrochemical means and the advantages associated with such functionalization in terms of practical utility.

## 2 Results and Discussion

### 2.1 General Aspects of the Cathodic Functionalization of 2H-Phase MoS_2_ with Organoiodides

**Figure 1:**
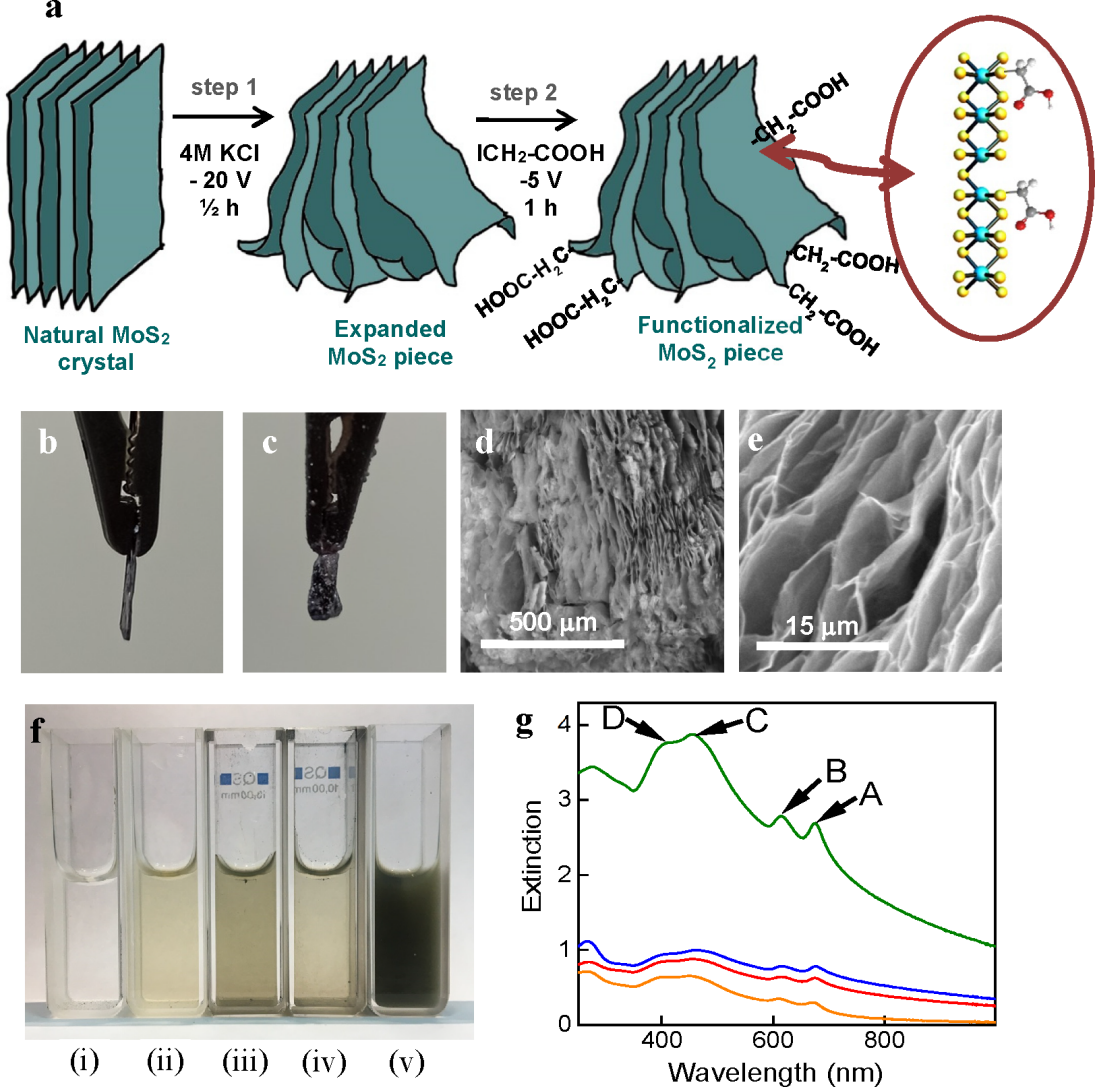
(a) Schematic of the electrochemical functionalization of 2H-MoS_2_, which consisted of two consecutive cathodic treatments: a natural MoS_2_ crystal was first expanded in aqueous KCl electrolyte (step 1) and then treated with iodoacetic acid (step 2). The inset illustrates the bonding of the acetic functionalities to sulfur atoms in areas of local metallic character of the 2H lattice, that is, close to sulfur vacancies and edges, as will be explained in section 2.3. (b, c) Digital photographs of the cross section of a MoS_2_ crystal (b) before and (c) after cathodic expansion in KCl. (d, e) Typical FE-SEM images of the cathodically expanded MoS_2_ material at different scales. (f) Digital photograph of the dispersions obtained by sonication in water of cathodically expanded MoS_2_ (i) without any subsequent treatment with iodoacetic acid or followed by an electrochemical treatment at −5 V with (ii) 0.25 M iodoacetic in water, (iii) 0.25 M iodoacetic in ethanol, (iv) 0.25 M iodoacetic in isopropanol, and (v) 0.05 M iodoacetic and 0.15 M Na_2_SO_4_ in water. (g) UV–vis extinction spectra of the aqueous dispersions displayed in part f: (i) black (null absorbance), (ii) orange, (iii) red, (iv) blue and (v) green traces. The excitonic bands A, B, C, and D, which are characteristic of 2H-phase MoS_2_, have been labeled for clarity.

Due to their simplicity and versatility, electrolytic methods constitute a very attractive tool for the exfoliation and functionalization of 2D materials,^20,21^ but such methods have so far remained underexplored, especially in the case of TMDs. Figure 1a shows a schematic representation of the protocol developed here to obtain functionalized 2H-phase MoS_2_ NSs, which consisted of two consecutive electrolytic steps, namely, (1) delamination of a bulk MoS_2_ electrode and (2) functionalization proper of the delaminated MoS_2_. Both steps were carried out in a two-electrode configuration using a piece of MoS_2_ as the working electrode and platinum foil as the counter electrode. The detailed experimental procedure is described in the Supporting Information (SI), but its main features are outlined in the following. First, based on an electrochemical exfoliation method that preserves the original 2H phase of the starting material and was recently reported elsewhere,^22^ a piece of natural MoS_2_ crystal was cathodically delaminated in aqueous KCl electrolyte (Figure 1a, step 1). Such an electrolytic treatment triggered expansion of the MoS_2_ crystal in an accordion-like fashion (Figure 1b and c). As a result, micrometer- and submicrometer-sized voids were generated between the delaminated MoS_2_ layers within the crystal [see the field emission scanning electron microscopy (FE-SEM) images in Figure 1d and e], but at the same time electrical contact of the delaminated layers with the electrode was preserved. Because access of both electrical current and electrolyte to the expanded layers should be guaranteed under such conditions, this initial cathodic treatment was expected to set a favorable stage for any subsequent electrochemical modification of the MoS_2_ material.^23^

We note that anodic exfoliation approaches documented in the literature for MoS_2_ tend to give expanded/delaminated products that easily detach from their parent electrode material,^24−26^ making them less amenable to serial electrolytic treatments. In contrast, other cathodic exfoliation processes generally afford expanded TMD materials that remain attached to the electrode as a single entity (i.e., very much like what is observed here in Figure 1c–e) but rely on the use of organic cations and noninnocuous organic solvents as the electrolytic medium.^27,28^ Here, expansion of the MoS_2_ electrode was accomplished in water with a widely available and inexpensive salt (KCl) and was therefore more attractive from a practical perspective.

Following electrolytic expansion, the MoS_2_ crystal was subjected to a second cathodic treatment in a solvent containing an organoiodide for the purpose of functionalization (Figure 1a, step 2). The main rationale behind this second step was that the iodine–carbon bond in the organoiodide should be readily cleaved under cathodic conditions, as it is well-known from prior organic electrosynthesis studies carried out mainly in polar aprotic solvents but also in water.^29,30^ Such a cathodic reaction could in principle give the iodide anion, I^–^, and the corresponding organic radical, •R, as described in eq 1:
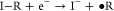
1

In turn, the generated organic radical could be expected to react with the 2H-phase MoS_2_ surface to afford a derivatized product. As a matter of fact, the grafting of organic (aryl and alkyl) moieties onto metal and carbon surfaces based on the cathodic reduction of their corresponding organoiodides has been previously shown to be feasible.^31,32^ We note that the organic radical can be further reduced to yield a carbanion, R^–^, as in eq 2:
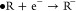
2, but it is known that this does not preclude the grafting of the organic moiety on the metal or carbon surface.^31,32^ In our case, iodoacetic acid was selected as the main benchmark reagent with a twofold purpose. Specifically, if the proposed strategy was to succeed in functionalizing the 2H-phase MoS_2_ NSs with acetic acid groups, we would expect the NSs to become hydrophilic and thus (i) to be readily dispersible in water, so that their aqueous dispersibility could be taken as a straightforward proxy for successful derivatization, and (ii) to exhibit enhanced performance in certain target applications, as will be discussed below.

Preliminary functionalization tests were accomplished by cathodic treatment of the expanded MoS_2_ electrode at −5 V for 60 min with 0.25 M iodoacetic acid in three different solvents: water, ethanol, and isopropanol. To probe the effect of the treatments on the aqueous dispersibility of the MoS_2_ NSs, the treated electrode was rinsed with water and dried under a vacuum, and then a weighed amount of it was transferred to neat water and bath-sonicated for 1 h to extract individual NSs. After allowing the sonicated product to stand undisturbed for 24 h or subjecting it to low-speed centrifugation, the resulting supernatant volume was collected and kept for subsequent analysis. It is well-known that nonfunctionalized 2H-phase MoS_2_ NSs, including those obtained by cathodic exfoliation, are not generally dispersible in aqueous medium by themselves.^22,33^ In fact, sonication of the as-expanded MoS_2_ crystal (i.e, the crystal that was just cathodically delaminated but not subjected to any subsequent treatment with iodoacetic acid) failed to give MoS_2_ dispersed in water in any detectable quantity. This was readily apparent from the fully transparent and colorless solution shown in Figure 1f(i) but was also confirmed by UV–vis absorption spectroscopy, which revealed virtually null absorbance in the whole wavelength range between 300 and 1000 nm (see black trace in Figure 1g).

On the other hand, the efficacy of a given cathodic treatment in derivatizing the MoS_2_ electrode with acetic acid groups should be reflected on the amount of MoS_2_ NSs that are retained in the aqueous supernatant after sonication, with larger dispersed amounts denoting higher derivatization abilities. Indeed, treatment of the expanded MoS_2_ electrode at −5 V with 0.25 M iodoacetic acid in the above-mentioned solvents led to solutions (after sonication in water) that exhibited a faint, although clearly visible, green tone, as noticed in Figure 1f(ii), (iii), and (iv) for the cathodic treatment in water, ethanol, and isopropanol, respectively. This result was consistent with the presence of 2H-phase MoS_2_ NSs dispersed in the aqueous medium,^12,22^ which in turn suggested that some functionalization of the expanded electrode was attained. Indeed, the features observed in the UV–vis extinction spectra of these aqueous solutions (orange, red, and blue traces in Figure 1g) completely agreed with those expected for 2H-phase MoS_2_, in particular the A, B, C, and D exciton peaks located at ∼675, 615, 456, and 411 nm, respectively.^34^ By contrast, cathodic treatment of the expanded electrode in the neat solvents (i.e., in the absence of iodoacetic acid) yielded colorless solutions after sonication that had null absorbance (spectra not shown), thus highlighting the central role played by the organoiodide in attaining water-dispersible MoS_2_. Furthermore, based on previously developed metrics,^35^ the UV–vis extinction spectra were used to estimate the concentration of MoS_2_ in their corresponding aqueous dispersions. Table 1 collects the concentrations determined for a set of functionalization trials, where it can be seen that cathodic treatment with 0.25 M iodoacetic acid in the three tested solvents afforded values around 10 mg L^–1^, being slightly lower for water. However, because working with the latter is preferable to using organic solvents on environmental and practical grounds, all further functionalization efforts were carried out in aqueous medium. Likewise, replacing iodoacetic acid by its nonhalogenated counterpart (i.e., acetic acid) led to cathodically treated MoS_2_ that could not be dispersed in water. This was consistent with the idea that stabilization of the MoS_2_ NSs relied mainly on the implantation of acetic acid radicals generated via reductive cleavage of the iodine–carbon bond of iodoacetic acid.

**Table 1:** Concentrations of MoS_2_ Aqueous Dispersions for a Set of Functionalization Trials^a^

**Reagent**	**Solvent**	**Supporting Electrolyte**	**Voltage (V)**	**[MoS**_**2**_**(aq)](mg L^–1^)**
0.25 M ICH_2_–COOH	water		–5	8
0.25 M ICH_2_–COOH	ethanol		–5	10
0.25 M ICH_2_–COOH	isopropanol		–5	12
0.25 M CH_3_–COOH	water		–5	0
0.25 M ICH_2_–COOH	water	0.15 M Na_2_SO_4_	–5	16
0.05 M ICH_2_–COOH	water	0.15 M Na_2_SO_4_	–5	45
0.01 M ICH_2_–COOH	water	0.15 M Na_2_SO_4_	–5	11
0.05 M ICH_2_–COOH	water	0.15 M Na_2_SO_4_	–2.5	26
0.05 M ICH_2_–COOH	water	0.15 M H_2_SO_4_	–5	20
0.25 M ICH_2_–COOH	water		–10	0
0.5 M ICH_2_–COOH	water		–10	11

a Concentrations of MoS_2_ aqueous dispersions, [MoS_2_ (aq)], obtained by sonication of a MoS_2_ electrode in water (nominal concentration: 2 mg mL^–1^) after cathodic expansion at −20 V in 4 M KCl aqueous solution followed by subsequent functionalization treatments in the specified conditions.

The above results suggested that cathodic treatment in the presence of only iodoacetic acid is associated to somewhat poor derivatization efficiencies, which were embodied in rather low dispersed MoS_2_ concentrations in water. To facilitate the functionalization reaction and attain larger amounts of derivatized NSs dispersed in water, a supporting electrolyte (Na_2_SO_4_) was added to the aqueous iodoacetic acid solution. Specifically, in the presence of 0.15 M Na_2_SO_4_, a higher dispersed concentration (about twice as large) could be achieved with 0.25 M iodoacetic acid. However, even higher MoS_2_ concentrations (∼45 mg L^–1^) were obtained by decreasing the amount of iodoacetic acid in the electrolytic solution to 0.05 M [see Table 1, Figure 1f(v), and the green trace in Figure 1g]. The latter appeared to be the optimum organoiodide concentration, since further reducing it to 0.01 M led to a substantial decrease in the final MoS_2_ concentration in water (see Table 1). Such an outcome implied that a limit in the extent of derivatization of the expanded MoS_2_ electrode was reached for iodoacetic acid concentrations around 0.05 M. The concentration of dispersed MoS_2_ also decreased noticeably when using smaller cathodic potentials (e.g., −2.5 V). From the results gathered in Table 1, the following cathodic treatment conditions were selected as the most efficient in terms of the amount of derivatized product that could be obtained: 0.05 M iodoacetic acid at −5 V with 0.15 M Na_2_SO_4_ as supporting electrolyte.

### 2.2 Physicochemical Characterization of the Cathodically Functionalized 2H-Phase MoS_2_ Nanosheets

**Figure 2:**
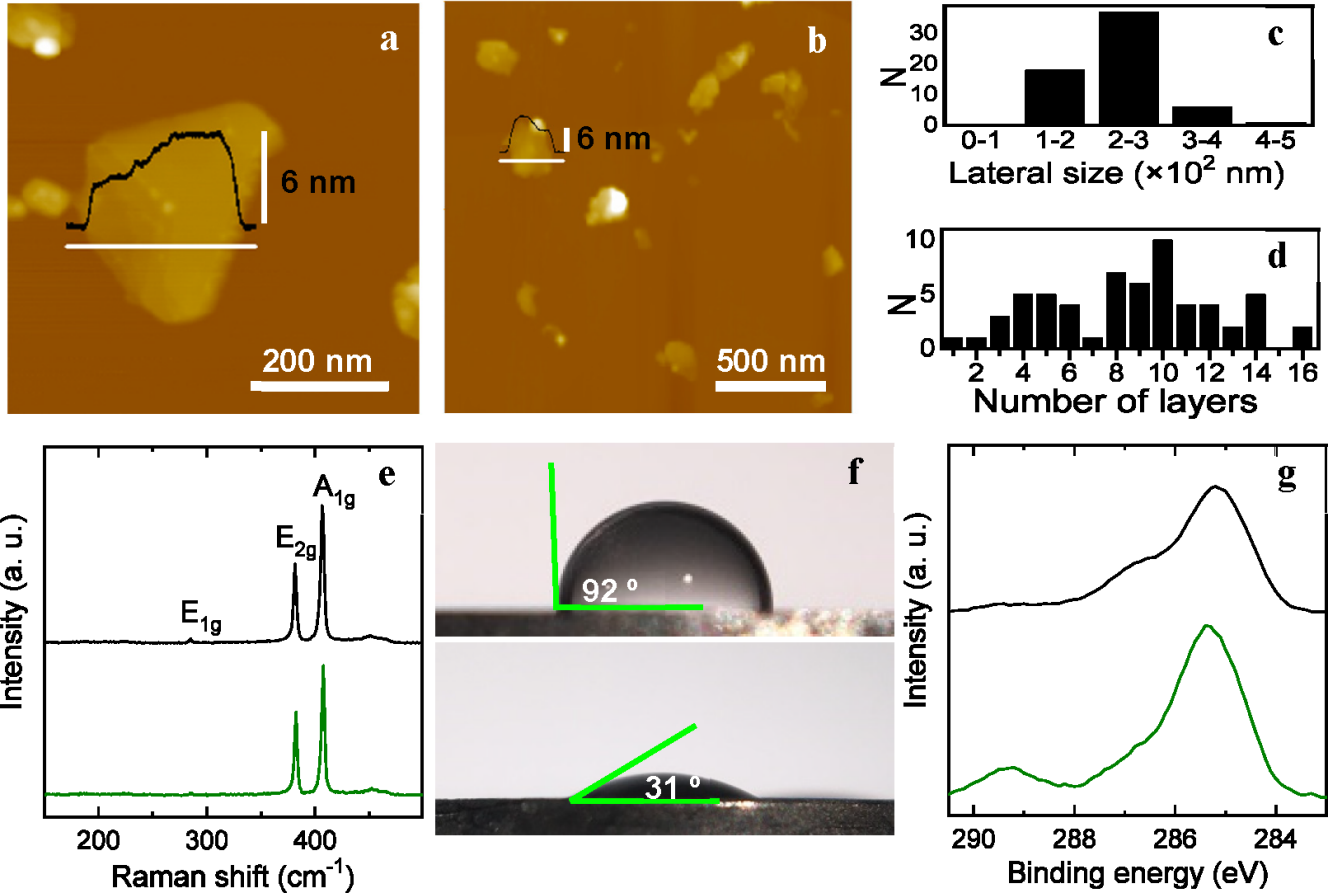
Characterization of aqueous dispersed, cathodically modified MoS_2_ flakes. (a, b) Representative AFM images of MoS_2_ flakes deposited from their dispersion. Representative line profiles (black lines) taken along the marked white lines are shown overlaid on the images. Histograms of (c) nanosheet lateral size and (d) layer number measured from the AFM images. (e) Raman spectrum of nanosheets extracted from as-expanded, nonfunctionalized MoS_2_ (black trace) and from functionalized MoS_2_ (green trace). The main bands are labeled for clarity. (f) Digital pictures of droplets of water on thin films of (top) nonfunctionalized and (bottom) functionalized MoS_2_ nanosheets, with indication of the contact angle. (g) High resolution C 1s core level XPS spectra of nonfunctionalized (black trace) and functionalized (green trace) MoS_2_ nanosheets.

Analysis of the aqueous dispersed, cathodically treated MoS_2_ material by atomic force microscopy (AFM; Figure 2a and b) revealed it to be comprised of NSs with typical lateral dimensions between one and several hundred nanometers as well as thickness in the 2–11 nm range, with the latter amounting to flakes that incorporated ∼1–16 monolayers. Histograms providing the distribution of NS lateral size and layer number from the AFM images are shown in Figure 2c and d, respectively. These data were in agreement with the average lateral size (∼300 nm) and layer number (∼10) of the dispersed MoS_2_ NSs as estimated from their UV–vis absorption/extinction spectral features.^35^ Significantly, such results were very similar to those obtained for NSs extracted by sonication from the as-expanded MoS_2_ electrode and dispersed in water and organic solvents with the aid of colloidal stabilizers or surfactants,^22^ indicating that the present cathodic functionalization treatment did not have any substantial impact on the morphology of the derivatized products. We note that 2H-phase MoS_2_ NSs can be made water-dispersible by themselves (i.e., without the need to use any colloidal stabilizers) when they are aggressively broken down into nanodots having lateral sizes of a few tens of nanometers or below,^36^ but such a scenario did not appear to be in place here. Furthermore, Raman spectroscopy (Figure 2e) indicated that the microscopic structure of the NSs was not significantly altered by the functionalization treatment, as evidenced by the virtually identical spectral features (A_1g_ and E^1^_2g_ bands characteristic of 2H-phase MoS_2_)^37^ noticed for NSs directly extracted from the as-expanded MoS_2_ electrode (black trace) and for NSs extracted from the cathodically derivatized electrode (green trace). We therefore concluded that the dispersibility of the latter in aqueous medium had to be related to changes in their surface chemistry (i.e., introduction of acetic acid groups on the NS surface) rather than to morphological and/or structural changes.

According to zeta potential measurements, the cathodically functionalized NSs were colloidally stabilized in water by the presence of negative electrical charges. Specifically, the zeta potential was determined to be about −41 and −25 mV at a pH of ∼10 and 4, respectively. These values were sufficient to endow the dispersed MoS_2_ NSs with some degree of colloidal stability based on electrostatic repulsions (even good stability at pH 10).^38^ Indeed, the corresponding dispersions were seen to remain stable (i.e., they remained visually homogeneous) at least for days or weeks. Under more acidic conditions (e.g., pH ∼2), the MoS_2_ dispersions were highly unstable, with the NSs sedimenting very rapidly, which made the zeta potential measurement unreliable. These results agreed with the negative charges in the NSs being mainly furnished by deprotonation of the carboxylic acid in the acetic acid groups. We assumed that the p*K*_a_ of the acetic acid groups grafted onto MoS_2_ was around 4, which was reasonable considering that the corresponding values for iodoacetic, acetic, and mercaptoacetic acids are ∼3.2, 4.8, and 3.6, respectively.^39^ The latter molecule was taken as a realistic approximation of the scenario found in the functionalized MoS_2_ NSs, as the acetic acid groups are believed to be bound to the NSs through their sulfur atoms (see below). Based on these considerations, we would expect the acetic acid groups in MoS_2_ to be (i) fully deprotonated at pH values well above 4, thus providing the NSs with their largest possible net negative charge and zeta potential (in absolute value), (ii) ∼50% deprotonated at pH 4, yielding NSs with zeta potential about half the magnitude of that measured at higher pH, and (iii) essentially nondeprotonated at pH below 4, so that the net charge and zeta potential of the dispersed NSs should approach zero, yielding highly unstable dispersions.^38^ Indeed, this expected trend was in agreement with the actual results from the zeta potential measurements and colloidal stability at the different pH values. Decoration of the MoS_2_ NSs with acetic acid groups should also be associated to them being more hydrophilic relative to their nonfunctionalized counterparts. While the ability to disperse the former in water was a clear indication of increased hydrophilicity, a more quantitative estimate was obtained from the measurement of water contact angles. The contact angles determined for thin films of nonfunctionalized and cathodically functionalized MoS_2_ NSs were, respectively, ∼92 and 31° (Figure 2f), which confirmed the improved hydrophilicity of the latter.

The presence of carboxylic acids on the cathodically derivatized NSs was corroborated by X-ray photoelectron spectroscopy (XPS). First, we note that upon cathodic treatment with iodoacetic acid, no sign of iodine could be detected in the survey spectrum of the MoS_2_ NSs (see Figure S1 in the Supporting Information). By contrast, while the high resolution C 1s core level spectrum of the nonfunctionalized material only exhibited the well-known adventitious carbon-related band located at ∼285 eV (Figure 2g, black trace),^40^ that of the cathodically functionalized material included an additional component at ∼289 eV (green trace), which is typical of carbon atoms from carboxylic acids.^41^ Hence, the appearance of a carboxylic acid signal together with the lack of iodine bands in the spectra was a strong indication of the successful functionalization of the MoS_2_ NSs with acetic acid groups. Additional evidence on functionalization obtained by ATR-IR is given in the SI of the manuscript (Figure S8). In addition, the high resolution Mo 3d and S 2p core level spectra of the nonfunctionalized and functionalized NSs (Figure S2a of the Supporting Information) provided evidence that the original 2H phase of the material was preserved upon the cathodic derivatization step. Specifically, the two peaks of the Mo 3d doublet band (3d_3/2_ and 3d_5/2_ peaks) appeared at virtually the same location in both the nonfunctionalized and functionalized NSs, namely ∼233 (3d_3/2_) and ∼229.8 (3d_5/2_) eV, and such a position is known to be characteristic of the 2H polymorph of MoS_2_ (the peaks of the 1T phase would appear downshifted by ∼0.8 eV).^12,28,42^ Similarly, the position of the S 2p doublet band, that is, ∼163.9 (2p_1/2_) and ∼162.7 (2p_3/2_) eV, remained unchanged after functionalization (Figure S2b) and was also consistent with 2H-phase MoS_2_.^42^

To estimate the extent of surface functionalization achieved by the present cathodic approach, XPS data were used to calculate the number of carbon atoms from carboxylic acids, which amount to the number of acetic acid groups, relative to the number of sulfur atoms located in the outermost atomic layer of the MoS_2_ NSs (we assume that acetic acid groups are only grafted on the very surface of the NSs, which is made up of a monolayer of sulfur atoms). The measurements were carried out on a piece of as-received, nonexfoliated MoS_2_ crystal that was just cathodically derivatized with iodoacetic acid, rather than on a thin film formed by the restacking of exfoliated and functionalized NSs. The reason behind such a modus operandi was the following. Because the MoS_2_ NSs produced by the present methodology were multilayered objects that exhibited a broad layer number distribution (see Figure 2d), it was not possible to accurately determine the fraction of surface sulfur atoms in the NSs out of the total number of sulfur atoms probed by the XPS technique for a thin film of restacked NSs. On the other hand, such a fraction could be reasonably assessed for a nonexfoliated, bulk MoS_2_ crystal, where cathodic derivatization was expected to take place only on its very surface. Here, the corresponding fraction of surface sulfur atoms out of the total sulfur probed by XPS could be readily gauged from the photoelectron emission cross section of MoS_2_ for 2p electrons from the S 2p core level as a function of emission depth (see SI for details). Specifically, the outermost sulfur atomic layer of the MoS_2_ crystal contributed ∼14% of the total sulfur signal (details of the calculation can be found in the SI). From this data and the calculated amount of carbon atoms from carboxylic acids derived from the C 1s spectrum of the functionalized crystal (Figure S3 of the Supporting Information), the degree of functionalization was estimated to be ∼0.10 acetic acid groups per surface sulfur atom. This figure can be compared with values in the range of ∼0.15–0.35 molecular groups per surface sulfur atom previously reported for (mainly monolayer) 1T-phase MoS_2_ NSs derivatized with different organoiodides.^8,9,43^ In general terms, the somewhat lower extent of derivatization observed here for the 2H-phase NSs was not surprising, considering that their semiconducting nature should make them less chemically reactive than their metallic 1T-phase counterparts.^7^ It is worth noting that cathodic functionalization could also be attained with other potentially useful organoiodides, which is exemplified here for the case of 4-iodoaniline. The derivatized NSs became dispersible in water as well (Figure S4a) of the Supporting Information, and again their analysis by XPS confirmed the incorporation of the key chemical group (amino group in this case; Figure S4b), together with the lack of any iodine (Figure S4c).

### 2.3 Rationalizing the Derivatization of 2H-Phase MoS_2_ Nanosheets with Organoiodides

While the results presented above indicated that 2H-phase MoS_2_ NSs can be functionalized with organoiodides by way of cathodic treatment, there still remains the question of how such a derivatization was actually made possible. In the case of 1T-phase MoS_2_ dispersed in water, the functionalization reaction is known to be spontaneous due to the presence of excess electrons in the NSs that can be readily transferred to the organoiodide, as in eq 1.^8,9^ These excess electrons are directly inherited from the preparation of the metallic 1T polymorph, which is generally based on chemical/electrochemical lithium intercalation processes,^44,45^ and thus are not to be found in the semiconducting 2H-phase counterpart, suggesting that reaction of the latter with organoiodides is not spontaneous. Such a hypothesis was corroborated from control experiments whereby a cathodically expanded MoS_2_ crystal was exposed to an aqueous 0.25 M iodoacetic acid solution, either under still conditions or under continuous sonication, in the absence of any applied voltage. In both instances, attempts to disperse the treated MoS_2_ material in water were unsuccessful; that is, the concentration of MoS_2_ in the final supernatant after sonication of the treated material in neat water was virtually zero. These results demonstrated that derivatization of the 2H-phase NSs with organoiodides needs to be triggered by an external supply of electrons, which in the present case took the form of a cathodic potential.

The external electrons required to prompt functionalization could also be sourced from a chemical species, for example, by resorting to a proper reducing agent. To illustrate this possibility, we selected sodium borohydride as the reductant, which is known to be able to cleave the iodine–carbon bond in organoiodides but cannot reduce carboxylic acids to aldehydes or alcohols.^46,47^ Thus, treatment of an expanded MoS_2_ crystal by sonication in an aqueous solution containing 0.25 M iodoacetic acid and 0.25 M sodium borohydride yielded a product that, after being rinsed, dried, and sonicated in neat water, afforded a non-negligible amount of dispersed MoS_2_ (∼15 mg L^–1^), as noticed from the corresponding photograph and UV–vis extinction spectrum (Figure S5) of the Supporting Information. By contrast, no dispersed MoS_2_ could be obtained at all when replacing iodoacetic acid by acetic acid in this experiment, which stressed the idea that NS functionalization relied on the generation of acetic acid radicals. Furthermore, we note that selected reducing agents (metallocenes) have been very recently used to boost the functionalization of 1T-phase MoS_2_ NSs with alkyl iodides (as well as other alkyl halides),^43^ thus constituting a similar strategy to that developed here for 2H-phase NSs with sodium borohydride. However, a key difference between both cases was that whereas a reductant was just employed to increase the extent of functionalization of the 1T-phase NSs with alkyl iodides (e.g., from ∼0.20 to 0.35 alkyl groups per sulfur atom), the reductant was strictly necessary to enable derivatization of the 2H-phase NSs. In summary, functionalization of both 1T- and 2H-phase MoS_2_ NSs with organoiodides requires an amount of extra electrons to be available. While the derivatization reaction is spontaneous for 1T-phase MoS_2_, it is not for 2H-phase MoS_2_, which is less reduced and thus needs an external electrochemical (or chemical) supply of extra electrons for the functionalization reaction to progress.

Another key issue to consider in the functionalization of 2H-phase MoS_2_ NSs with organoiodides concerns the type of interaction that is established between the NSs and the implanted moieties. It was apparent that the latter were not simply physisorbed on the MoS_2_ surface, since we noticed that both iodoacetic acid and acetic acid failed to act as aqueous colloidal stabilizers for 2H-phase NSs, obtained either by electrochemical exfoliation of MoS_2_ crystals or by direct exfoliation of MoS_2_ powder via sonication. Furthermore, the NSs that were cathodically derivatized with iodoacetic acid and then dispersed in water could be sedimented by centrifugation and easily redispersed in neat water as many times as desired, which constituted a strong indication that the acetic acid moieties were tightly bound to the NS surface (if the acetic acid groups were just weakly adsorbed, this procedure would have led to their progressive removal from the NS surface and thus to a loss of colloidal stability). Taking again the 1T-phase MoS_2_ NSs as a reference system, we note that the grafting of organic radicals from organoiodides has been previously shown to proceed in that case through formation of covalent bonds with the NS sulfur atoms (i.e., carbon–sulfur bonds).^8,9^ However, such a scenario is not immediately applicable to the case of 2H-phase NSs. The metallic character of the 1T polymorph implies that it possesses a non-negligible amount of (both occupied and empty) electronic states close to the Fermi level, which are absent from its semiconducting 2H counterpart.^7^ Such electronic states are expected to endow the former with increased chemical reactivity compared with 2H-phase MoS_2_. Indeed, theoretical work has demonstrated that attachment of alkyl and other radicals on the (pristine) MoS_2_ surface is energetically very favorable in the 1T polymorph but not in the 2H one.^7^

Nonetheless, the presence of certain defects in the 2H-phase MoS_2_ lattice is known to locally change its electronic structure from semiconducting to metallic (increased density of electronic states around the Fermi level). This is particularly the case not only of edge defects,^48^ which are relatively abundant in submicrometer-sized NSs, but also of sulfur vacancies.^49^ The latter are quite prevalent in bulk and 2D MoS_2_ of both natural and synthetic origin,^50,51^ given that sulfur vacancies with one or two absent atoms possess the lowest energies of formation among all defects.^51^ Such low formation energies render the introduction of sulfur vacancies easy during synthesis and processing of MoS_2_ materials. Furthermore, as the removal of a sulfur atom from the MoS_2_ lattice (yielding MoS_2–*x*_) is formally a reduction process, sulfur vacancies can be created by reaction with a sufficiently strong reducing agent^52^ or by electrochemical reduction.^53^ Thus, their generation can be expected to be favored under the intrinsically reductive conditions of the present cathodic treatments. The local metallic character of the 2H lattice around sulfur vacancies and edges in MoS_2_ should thus provide a number of sites with increased reactivity where organic radicals and other species can covalently attach to sulfur atoms, as recently shown for the grafting of aryl radicals from diazonium salts^15^ and for the interfacial bonding with nonlayered chalcogenides.^54^ Both possibilities, that is, acetic groups bonding to sulfur atoms on the edges of the layers and near sulfur vacancies, are illustrated in the inset to Figure 1a. We note that functionalization does not deactivate the sulfur vacancies, as it does not take place in the vacancy itself (the undercoordinated molybdenum) but in its surroundings (sulfur atoms nearby). Thus, molybdenum atoms which lack sulfur remain unsaturated after functionalization. This is demonstrated by the detection of a well-defined, significant signal associated to the presence of Mo–S dangling bonds in the electron paramagnetic resonance (EPR) spectrum of the functionalized MoS_2_ material (see Figure S9 in the SI). A typical vacancy passivation method would involve reaction with alkanethiol molecules,^55^ which would provide extra sulfur atoms to fill the vacancy. The grafting of the acetic groups to sulfur atoms in MoS_2_ layers is indeed suggested by the weak IR absorption observed at ∼630 cm^–1^ in the ATR-IR spectrum of functionalized MoS_2_, which is ascribable to C–S stretching^8^ (see Figure S8 of the SI). The formation of carbon–sulfur bonds in the cathodically functionalized NSs could in principle be disclosed by XPS as well through the emergence of a doublet component (S–C component) in the S 2p spectrum at ∼164 eV (2p_1/2_) and 163 eV (2p_3/2_).^8,9^ However, in the present case such a component could not be clearly detected for two main reasons: (1) The multilayered nature of the MoS_2_ NSs implies that the percentage of (surface) sulfur atoms that are covalently bonded to carbon must be very small. From the degree of derivatization with iodoacetic acid determined above, the relative weight of the S–C component in the S 2p spectrum was estimated to be just a very few percent, compared to ∼20% for functionalized 1T-phase MoS_2_ monolayers.^9^ (2) The S–C component overlaps significantly with the component associated to the pristine 2H phase, although less so with that of the 1T phase. In fact, even for monolayer MoS_2_, functionalization has been very recently reported to introduce only subtle changes in the XPS S 2p core level spectrum.^18^ Both factors contributed to making the S–C component in the functionalized 2H-phase NSs virtually unnoticeable (Figure S2b). (As a side note, a signal at a binding energy very close to that of the S–C component was occasionally seen in the spectra; this signal arose from the presence of bismuth, which is a common impurity in natural MoS_2_ crystals; see Figure S6 of the SI). On the other hand, carbon–sulfur bonds can also be detected in the C 1s spectrum through a C–S component that would be located at about 285.5 eV.^56^ Although this component overlaps with the signal arising from adventitious carbon, its presence could be readily brought to light by subtracting the normalized C 1s spectrum of the nonfunctionalized NSs from that of their functionalized counterpart, the result of which is shown in Figure S7 of the SI. In addition to the expected component at about 289 eV associated to carboxylic acids, a new component at ∼285.5 eV emerged in the differential spectrum, which could be ascribed to carbon–sulfur bonding. This result was thus consistent with the acetic acid groups derived from iodoacetic acid being covalently bonded to sulfur atoms from the MoS_2_ NSs.

Finally, we note that during the cathodic treatment of MoS_2_ with organoiodides, other types of reaction can be expected to compete with the functionalization reaction proper. Because the derivatization process was carried out in water, this should be the case of the hydrogen evolution reaction (HER), which is known to be catalyzed by MoS_2_, especially under acidic conditions.^57^ Indeed, when Na_2_SO_4_ was replaced by H_2_SO_4_ as the supporting electrolyte in the cathodic treatment with iodoacetic acid, a substantial decrease in the amount of MoS_2_ dispersed in water was observed (Table 1). Moreover, in the absence of a supporting electrolyte, the application of larger cathodic potentials (e.g., −10 V), which should trigger a more vigorous HER, failed to give any dispersed MoS_2_, although this outcome could be reversed if the concentration of iodoacetic acid was also increased (e.g., from 0.25 to 0.50 M). Such results can be rationalized bearing in mind that edges and sulfur vacancies are the main catalytic active sites for HER on the surface of 2H-phase MoS_2_,^53,57−59^ that is, the very same sites that are thought to be reactive toward organoiodide functionalization. Consequently, a competition for these sites should be at work between the derivatization reaction and the HER, and so any change in the cathodic treatment conditions that favors the latter should negatively impact the former. It can also be argued that hydrogen atoms adsorbed on MoS_2_ vacancies/edges, as an intermediate species of the HER, will react with the cathodically reduced organoiodide to give the corresponding organic molecule (e.g., acetic acid in the case of iodo acetic acid), thus preventing the derivatization of the MoS_2_ surface. While this reaction might be taking place to some extent, we believe that it will be significantly inhibited by the generation of molecular hydrogen from the hydrogen adatoms via the Tafel and/or Heyrovsky steps of HER, which is favored at the catalytic sites of MoS_2_.^57^ The promoted generation of molecular hydrogen should then give the organic radicals derived from the organoiodide the opportunity to react with the MoS_2_ surface.

### 2.4 Application of Cathodically Derivatized 2H-Phase MoS_2_ Nanosheets in the Catalytic Reduction of Nitroarenes and Organic Dyes

The reduction of nitroarenes and organic dyes is of practical interest in the areas of environmental remediation and chemical synthesis. For example, a number of nitroarenes, such as 4-nitrophenol (4-NP) and 2-nitroaniline (2-NA), as well as organic dyes, including methyl orange (MO) and methylene blue (MB), are found in the wastewater effluents of the pesticide, herbicide, or pigment industries. These compounds frequently possess a highly toxic and recalcitrant character. Hence, they must be degraded before the effluents are released into the aquatic environment, with their reduction into more benign forms being a viable technological solution (e.g., anilines derived by nitroarene reduction are biodegradable and exhibit a lower toxicological profile).^60,61^ Also, the reduction of certain nitroarenes into their corresponding anilines constitutes one of the main steps in the synthesis of relevant pharmaceutical drugs (e.g., paracetamol) and polymers, such as Kevlar.^60,61^ While nitroarene/dye reduction has traditionally relied on precious metals (Pt, Pd, Ag, etc.) to catalyze the reaction, the need to deploy more earth-abundant and affordable catalysts has driven recent efforts on the use of non-noble metal-based systems.^60^ In this context, 2D MoS_2_ has demonstrated catalytic activity toward reduction reactions in water with sodium borohydride as the reductant, with the 1T-phase NSs being quite efficient in this role (probably due to their intrinsically metallic nature).^62,63^ Still, issues related to the structural instability/degradation of 1T-phase MoS_2_ mentioned above are an obstacle to its practical implementation. 2H-phase MoS_2_ NSs can also be used as reduction catalysts, but so far their activity has considerably lagged behind that of their 1T-phase counterparts.^52,64^ Thus, strategies that boost the performance of the 2H-phase NSs would be highly desirable. We show in the following that the cathodic functionalization approach developed here is one such effective strategy.

**Figure 3:**
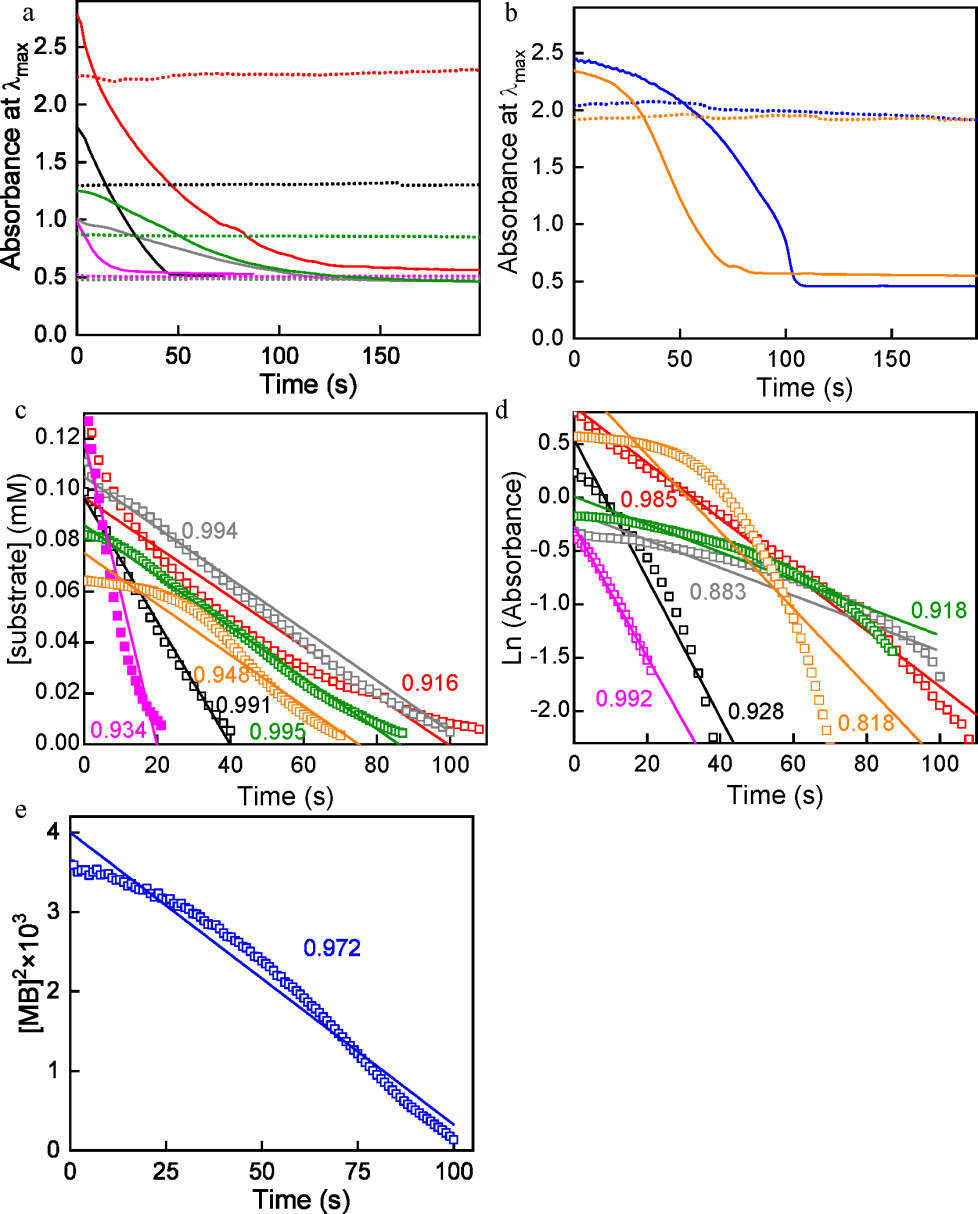
(a, b) Typical kinetic profiles (solid lines) measured for the reduction of different substrates of NaBH_4_ using functionalized MoS_2_ nanosheets as catalyst. The kinetic profiles were obtained by monitoring the absorbance at the wavelength of an absorption maximum of the substrate molecule (λ_max_). (a) Nitroarenes: 4-NP (red trace, λ_max_ = 400 nm), 2-NP (magenta, 416 nm), 4-NA (black trace, 380 nm), 2-NA (gray trace, 410 nm), nitrobenzene (green trace, 270 nm). (b) Organic dyes: MO (orange trace, 461 nm) and MB (blue trace, 675 nm). The corresponding blank experiments (dotted lines), that is, experiments where no catalyst was added, are included for comparison. (c–e) Fitting (solid line) of the experimental kinetic profile data (empty squares) to different orders of reaction with respect to the substrate: (c) first order, that is, exponential decay dependence of reaction rate with time; (d) zero order, that is, linear dependence; and (e) minus one order. For clarity, the corresponding regression coefficients R^2^ for the fittings are indicated using the same color code.

The catalytic tests were carried out in aqueous medium at room temperature with the nitroarenes 4-NP, 2-nitrophenol (2-NP), 4-nitroaniline (4-NA), 2-NA, and nitrobenzene (NB), and with the organic dyes MO and MB, using the acetic acid-functionalized 2H-phase MoS_2_ NSs as the catalyst. To allow direct comparisons with other catalysts reported in the literature, as well as to facilitate kinetic analyses of the reactions, typical substrate concentrations were chosen to be in the 0.06–0.12 mM range and the sodium borohydride reductant was used in a large excess relative to the substrate (see Experimental Section in the SI for details). In all cases, the reaction progress was followed with UV–vis absorption spectroscopy, by monitoring the intensity of an absorption peak that was characteristic of the substrate molecule (in the presence of the reducing agent) but was absent from its reduced counterpart (see Figure S10 of the SI for the corresponding spectra). Thus, according to the Beer–Lambert law, a change in the concentration of the substrate molecule as a result of its reduction should induce a directly proportional variation in the intensity of its absorption peak, which provided the basis for recording kinetic profiles for these reactions from the absorbance data. More specifically, kinetic profiles of the reactions were obtained by plotting the evolution of absorbance measured at 400, 416, 382, 410, 270, 461, and 675 nm for 4-NP, 2-NP, 4-NA, 2-NA, NB, MO, and MB, respectively. Figure 3 (solid lines) shows typical kinetic profiles recorded for the nitroarenes (a) and the organic dyes (b). Profiles corresponding to blank experiments, which monitored aqueous solutions that contained a given substrate and the reductant but lacked any catalyst, are also given in Figure 3 (dotted lines). As expected, the latter revealed time-invariant absorbance for all the tested nitroarenes and dyes, indicating that the molecules could not be reduced in the absence of a proper catalyst.

On the other hand, efficient substrate conversion was observed for all the nitroarenes and dyes in the presence of the acetic acid-functionalized 2H-phase NSs, as noticed by the sharp decrease of absorbance in their corresponding kinetic profiles. A plateau having nonzero absorbance was seen to develop upon reaction completion in the kinetic profiles (Figure 3). Rather than arising from unreacted substrate molecules, the finite absorbance associated to the plateaus was mostly due to the MoS_2_ catalyst itself. Indeed, the absorbance values measured for the latter at the concentration used in the reaction medium (∼7 mg L^–1^) were very similar to those recorded for the plateaus (e.g., ∼0.6 at 400 nm in the case of 4-NP; see Figure S11 of the SI for the extinction spectrum of the catalyst at 7 mg L^–1^). This observation indicated that essentially full conversion of the substrate molecules was attained in their reduction with the acetic acid-functionalized MoS_2_ catalyst.

To provide a quantitative measure of catalytic performance, the kinetic profiles were used to calculate the number of moles of substrate converted per unit time per mole of MoS_2_ in the reaction medium, which was taken as a direct proxy of catalytic activity. The resulting values for the different nitroarenes and dyes are collected in Table 2 and compared in Tables S1 and S2 of the Supporting Information with those reported in the literature for other MoS_2_- and non-noble metal-based catalysts. For example, a catalytic activity value of 63 h^–1^ was determined for the reduction of 4-NP, which could be directly weighed against a large pool of data available for this reaction (see Table S1; 4-NP reduction is a model reaction for the testing of catalytic systems in aqueous medium).^65^

**Table 2:** Catalytic Activity of the Acetic Acid-Functionalized 2H-Phase MoS_2_ Nanosheets^a^

**Substrate**	**Catalytic activity (h**^**–1**^**)**
4-nitrophenol	63
2-nitrophenol	340
4-nitroaniline	180
2-nitroaniline	90
nitrobenzene	78
methyl orange	71
methylene blue	44

a Catalytic activity calculated as the number of moles of substrate converted per unit time per mole of MoS_2_ catalyst in the reaction medium.

Good colloidal stability in the aqueous catalysis medium must be an important asset of the current functionalized MoS_2_ material, as it ensures that the NSs will not aggregate and thus the active catalytic sites on their surface will remain accessible for catalysis. Anyway, comparing with other previously reported colloidally stabilized MoS_2_ NSs, the catalytic activity was ∼3 times larger than that obtained with (nonfunctionalized) 2H-phase NSs produced by the present cathodic exfoliation method and dispersed in water with the aid of a biomolecular stabilizer [guanosine monophosphate (GMP)]^22^ and of the order of 10 times larger than that of NSs prepared by direct exfoliation of MoS_2_ via sonication in aqueous GMP solution.^66^ We believe the much improved performance of the acetic acid-funtionalized NSs to be due to three main factors: (1) the intrinsically reductive conditions of the cathodic exfoliation/derivatization processes should favor the generation of sulfur vacancies on the NSs,^53^ as was indeed the case (see Figure S9 in the SI) which are known to be highly active catalytic sites toward nitroarene/dye reduction (see the proposed reaction mechanism in subsection 3.4 and Figure S12 in the SI);^65^ (2) compared with GMP, the relatively small size of the acetic acid moieties present on the MoS_2_ NSs is expected to be associated to lower steric barriers for reagent access to the catalytic active sites, and (3) while GMP adsorbs preferentially at sulfur vacancy sites on the MoS_2_ surface due to specific interactions of acid–base type between its nucleobase moiety and the vacancy,^66^ the current functionalization takes place through sulfur atoms near the unsaturated molybdenum in edges and sulfur vacancies, but not on the vacancy itself (see inset to Figure 1a), and thus leaves the active sites unaffected and available for catalysis (see additional details in subsection 3.4 of the SI). The latter factor implies that, while colloidal stabilization with GMP as dispersant takes place at the expense of catalytic activity, the present functionalization strategy does not present such a drawback. Other strategies to boost the catalytic activity of 2H-phase MoS_2_ NSs, including inserting them within the galleries of montmorillonite,^64^ have met with considerable success, but the present approach clearly outperformed all of them (see Table S1). The acetic acid-functionalized 2H-phase NSs also outperformed 1T-phase MoS_2_ (obtained either by the lithium intercalation/exfoliation route or by hydrothermal synthesis), as well as most reported catalysts based on non-noble metals (e.g., Cu, Co, or Ni). Likewise, the cathodically derivatized NSs compared very favorably with other documented catalysts in the reduction of the other nitroarenes and the organic dyes (see Table S2). It was therefore concluded that the present functionalization strategy affords enhanced and highly competitive MoS_2_ catalysts.

The experimental kinetic profiles recorded for nitroarene and dye reduction with different catalysts usually obey either first- or zero-order behavior with respect to the substrate;^52,65^ that is, they obey one of the two following equationsReaction order of 1:

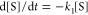
3Reaction order of 0:

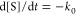
4where [S] is the substrate concentration and *k*_1_ and *k*_0_ are the apparent reaction rate constants. Because the reductant was used in a large excess relative to the substrate, its concentration was assumed to remain roughly constant throughout the reaction, and so its contribution to the rate equations was not explicitly included as it was implicitly incorporated in the *k*_1_ and *k*_0_ rate constants. Even though the catalytic reduction of nitroarenes/dyes is extensively investigated, the specific factors that govern its kinetic behavior and the corresponding reaction order are not usually known. As a first step to rationalize the kinetics, we note that in the presence of a large excess of reducing agent, the reaction rate should be mainly limited by the rate of diffusion of the substrate molecules to the catalyst surface.^67^ Within this framework, we hypothesized that the reaction kinetics for the present acetic acid-functionalized MoS_2_ catalyst was largely determined by the net electric charge of the substrate molecule. Such a hypothesis was based on the following reasoning: In the basic medium of the reduction reaction (pH ∼ 11 generated by the presence of sodium borohydride), the functionalized NSs are negatively charged, as discussed above; If the substrate molecule is negatively charged as well, its access to the catalyst will be hampered by an electrostatic repulsion barrier. Under such a scenario, the reaction rate can be expected to positively correlate with the substrate concentration; that is, the higher the substrate concentration, the higher the probability that a substrate molecule will be able to reach a catalytic active site, and thus the higher the reaction rate. This behavior would be better described by eq 3 (reaction order of 1). In our case, this situation would be in place for 4-NP, 2-NP, and MO, which are negatively charged in the reaction medium (the p*K*_a_ of both 4-NP and 2-NP is around 7, and that of MO is around 3.5).^39^

On the other hand, electrostatically unimpeded access to the catalyst is to be expected in the case of electrically neutral substrate molecules. As a result, the catalytic active sites should be more likely to become saturated with the substrate, so that the reaction rate will be much less sensitive to its concentration and hence will more probably obey eq 4 (reaction order of 0). 4-NA, 2-NA, and NB are electrically neutral in the basic reaction medium (the p*K*_a_ of the conjugate acid of 4-NA and 2-NA is around 1 and 0, respectively).^39^ Finally, a special situation may arise when the substrate molecule is positively charged (MB in our case). Here, a diffuse layer of substrate molecules should form on the surface of the MoS_2_ NSs by electrostatic attraction,^68^ which can be expected to hinder transport of the reactants and reaction products between the bulk of the solution and the catalytic active sites. Accordingly, the reaction rate should negatively correlate with the substrate concentration: as the latter is decreased, less compact diffuse layers should develop, which in turn should make reactant/product transport easier and lead to higher reaction rates. Hence, the corresponding kinetic behavior could be well described by a reaction order of −1 as reflected in the following equationReaction order of −1:

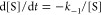
5

If the above reasoning is correct, eqs 3, 4, and 5 predict that the kinetic profiles of the reactions must adhere to exponential, linear, and square-root decay functions for negatively charged, neutral, and positively charged substrate molecules, respectively. To probe this, the experimental kinetic profiles (Figure 3a and b) were fitted to such types of decay function and the quality of the fits was assessed from their regression coefficients (R^2^). Figure 3c and d shows the results of the linear and exponential fits, respectively, for 4-NP, 2-NP, 4-NA, 2-NA, NB, and MO, with the corresponding R^2^ values also indicated in the plots. MB was not included because it was obvious (Figure 3b) that its kinetic profile could not be described by either a linear or exponential decay function. The quality of the fits was good (R^2^ ∼ 0.99) in the linear case with 4-NA, 2-NA, and NB, and in the exponential case with 4-NP and 2-NP, but it was poor (R^2^ < 0.94) in the linear case with 4-NP and 2-NP, and in the exponential case with 4-NA, 2-NA, and NB. Moreover, for MB a reasonable fit to a square-root decay function (R^2^ ∼ 0.97) was attained (Figure 3e). In the specific case of MO the fit was not good either in the linear case or in the exponential case. Given that MO reduction yields two distinct aniline derivatives as the reaction products (see Figure S10 in the SI),^69^ one neutral and the other negatively charged, their presence in the reaction medium is expected to result in a mixture of zero and first order kinetics, which explains why the profiles do not fit to either linear or exponential decay. Thus, overall, the results of the fittings of the experimental kinetic profiles agreed with the above prediction of the kinetic behavior with the present MoS_2_ catalyst being largely determined by the substrate charge. We note, however, that while the latter can be used as a proxy to understand the kinetics of the catalytic reduction, careful consideration of the specific characteristics of the catalyst should always be taken. For instance, the formation of a diffuse layer of positively charged substrate molecules on a catalyst that is negatively charged by weakly adsorbed (physisorbed) anionic surfactant molecules will probably be compromised by molecular exchange of the latter for the substrate molecules. Hence, the corresponding reduction kinetics will likely not be described by a reaction order of −1, as recently observed for MB reduction with MoS_2_ NSs stabilized by an anionic dispersant.^52^ In the present case, a robust diffuse layer is expected to form because the anionic acetic acid groups are strongly bound to the MoS_2_ NSs.

**Figure 4:**
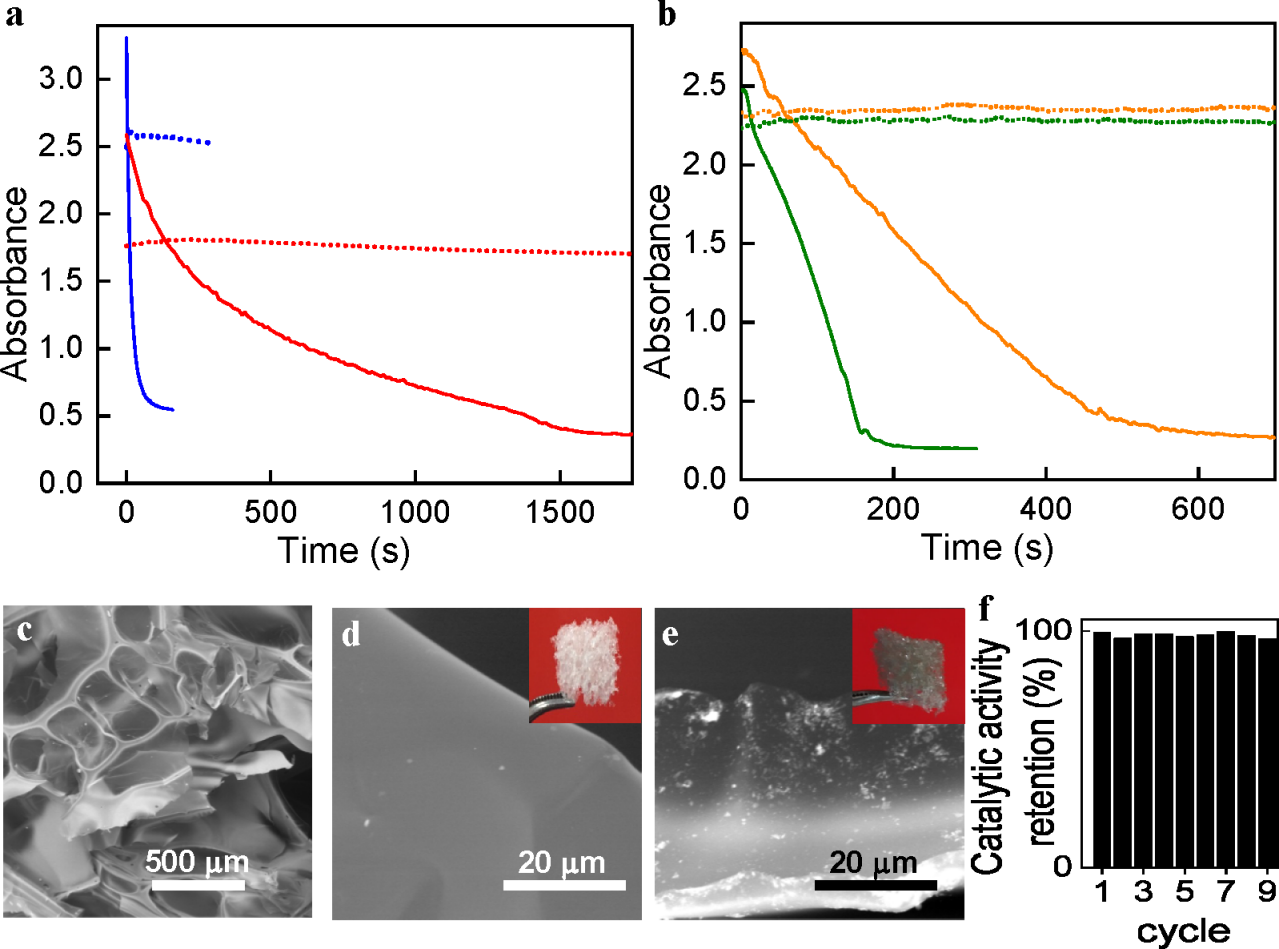
(a) Kinetic profiles for the reduction of 4-NP catalyzed by functionalized MoS_2_ nanosheets (catalyst concentration: 7 mg L^–1^) carried out in concentrated reaction media, namely: 1.2 mM 4-NP (λ = 455 nm, blue trace) and 12 mM 4-NP (λ = 479 nm, red trace). Blank experiments for each profile are also shown as dotted lines. (b) Kinetic profiles for the reduction of mixtures of nitroarenes catalyzed by functionalized MoS_2_ nanosheets (catalyst concentration: 3.5 mg L^–1^), namely: binary mixture of 4-NA (0.12 mM) and 2-NA (0.24 mM) (orange trace), and a ternary mixture of 4-NP (0.06 mM), 4-NA (0.06 mM), and 2-NA (0.12 mM) (gray trace). Blank experiments for each profile are also shown as dotted lines. (c–e) FE-SEM micrographs of polyurethane foam before (c, d) and after (e) coating with MoS_2_ nanosheets. Insets to d and e: digital photographs of cylinders ∼1 cm in diameter and ∼1 cm in height of the foam before and after coating, respectively. (f) Reusability experiments of polyurethane foam-supported MoS_2_ nanosheets in the catalytic reduction of 4-NP with NaBH_4_ ([4-NP] = 0.12 mM).

Finally, with a view to the practical implementation of the functionalized MoS_2_ catalyst, a number of further issues were considered. First, although the substrate concentrations used here (in the range of 0.1 mM) are quite convenient for the testing and comparison of catalyst performance, actual wastewater effluents usually contain much higher concentrations of the pollutants (∼10 mM).^70^ To test the ability of the functionalized NSs to work in more concentrated reaction media, the reduction of 4-NP was carried out at concentrations ten and a hundred times higher (i.e., 1.2 and 12 mM). The resulting kinetic profiles are presented in Figure 4a. An exponential decay function (reaction order of 1, eq 3) was still observed for both concentrations, which means that the substrate concentrations are not still sufficiently high to saturate the active sites of the catalyst. In fact, the time required to reaction completion with a concentration ten times higher than that originally tested (1.2 mM 4-NP) is more or less the same, and thus the catalytic activity is ten times higher (660 h^–1^ at 1.2 mM vs 63 h^–1^ at 0.12 mM 4-NP). With a difference of another factor of 10 (12 mM 4-NP), the time required to reaction completion was ∼10 times higher so that the catalytic activity remained similar (∼600 h^–1^), which indicated that the catalytic performance of the NSs was not impaired by the presence of a large amount of substrate. Second, the catalyst was also efficient in the reduction of nitroarene mixtures, which can be present in industrial wastewaters, as shown in Figure 4b for the kinetic profiles of a binary (4-NA, 2-NA) and a ternary (4-NA, 2-NA, 4-NP) mixture. The measured catalytic activities (∼370 and 75 h^–1^ for the binary and ternary mixtures, respectively) were in the range of those determined for the corresponding single substrates (see Table 2). Third, immobilization of the catalyst on a proper substrate can be a convenient way to facilitate its handling and reuse. The functionalized MoS_2_ NSs could be immobilized on a polyurethane foam scaffold by dipping the latter into an aqueous NS solution that was then allowed to dry. Figure 4c–e shows FE-SEM images and digital pictures of the foam before (c and d) and after (e) NS coating. The cellular, macroporous structure of the polyurethane foam is clearly appreciated in the lower resolution image (Figure 4c). Testing of the immobilized catalyst toward 4-NP reduction indicated that it could be used for nine consecutive cycles without experiencing a large decline in activity (Figure 4f).

## 3 Conclusions

We have demonstrated that 2H-phase MoS_2_ NSs can be functionalized with molecular groups derived from organoiodides by a straightforward and expeditious electrolytic method. Such a method relies on the cathodic treatment of a previously expanded MoS_2_ crystal in an electrolyte that contains the organoiodide (iodoacetic acid or 4-iodoaniline), yielding a derivatized material that, contrary to its nonfunctionalized counterpart, can be colloidally dispersed in aqueous medium. Changes detected in the surface chemistry, water contact angle, and zeta potential of the MoS_2_ NSs confirmed their successful molecular functionalization. The derivatization reaction was not spontaneous and required an external supply of electrons to proceed, which in the present case originated from the application of a cathodic potential but could be obtained from a reducing agent as well. Grafting of the molecular groups on the 2H-phase MoS_2_ NSs was also thought to be made possible by the locally enhanced chemical reactivity associated with intrinsic lattice defects, especially sulfur vacancies. The acetic acid-functionalized NSs exhibited a high catalytic activity in the reduction of nitroarenes and organic dyes, which is of practical relevance for the treatment of industrial wastewater effluents, outperforming most (if not all) 1T- and 2H-phase MoS_2_ and other non-noble metal-based catalysts previously investigated for this purpose. The functionalized MoS_2_ catalyst retained most of its activity even at the high reactant concentrations typical of actual wastewater effluents, also performed efficiently in the reduction of binary and ternary mixtures of the reactants, and could be immobilized onto a polymer scaffold to facilitate its manipulation and reuse. Further, a correlation between the kinetic behavior of the reduction reaction (i.e., reaction order) and the net electric charge of the reactant molecule was established and rationalized on the basis of the relative ability of the latter to diffuse to the active sites of the catalyst. Finally, we note that the molecular derivatization strategy developed here should be beneficial for the application of 2H-phase MoS_2_ NSs in areas beyond catalysis. For instance, biomolecules, polymers, and other nanostructures could be covalently attached to the functionalized NSs through their carboxylic acid or amino groups, yielding hybrid materials that could find practical use in, for example, biomedicine or energy conversion and storage.

## Supporting Information Available

Experimental section, additional characterization of the functionalized MoS_2_ materials, and additional information on the performance of functionalized MoS_2_ NSs as catalysts (PDF)
