# Supplementary material for: Molecular Functionalization of 2H-Phase MoS2 Nanosheets via an Electrolytic Route for Enhanced Catalytic Performance
Source: ACS Appl Mater Interfaces. 2021 Jul 12;13(28):33157–71. doi: 10.1021/acsami.1c08850 (PMC8397248; doi:10.1021/acsami.1c08850)
Supplement: Supplementary file 1 — am1c08850_si_001.pdf [file am1c08850_si_001.pdf]

Supporting Information

Molecular functionalization of 2H-phase MoS_2 nanosheets via an electrolytic route for enhanced catalytic performance

S. García-Dalí, J.I. Paredes*, S. Villar-Rodil*, A. Martínez-Jódar, A. Martínez-Alonso, J.M.D. Tascón

Instituto de Ciencia y Tecnología del Carbono, INCAR-CSIC, Francisco Pintado Fe 26, 33011 Oviedo, Spain

*Corresponding authors' e-mail addresses:
paredes@incar.csic.es (J. I. Paredes), silvia@incar.csic.es (S. Villar-Rodil).

1 Experimental section

1.1 *Materials and reagents*

Pieces of MoS_2 natural crystals (approximate dimensions: ~10×10×0.5 mm^3) were obtained from SPI Supplies. Platinum foil (25×25×0.025 mm^3) was purchased from Good Fellow. Ethanol was obtained from VWR. H_2SO_4 25 %, KCl (>0.005% Br), isopropanol, iodoacetic acid (purity ≥98.0%), $NaBH_4$, 4-iodoaniline (≥98.0%), 4-nitrophenol (4-NP), 2-nitrophenol (2-NP), 4-nitroaniline (4-NA), 2-nitronailine (2-NA), nitrobenzene (NB), methylene blue (MB), and methyl orange (MO) were obtained from Sigma-Aldrich and used as received. Milli-Q deionized water (Millipore Corporation; resistivity: 18.2 MΩ•cm) was employed in all the experiments.

1.2 *Electrochemical functionalization*

The functionalization of MoS_2 was carried out in two consecutive electrochemical steps, both performed in cathodic conditions and in a two-electrode set-up. A ~5×5×0.5 mm^3 piece of MoS_2 was first cut from the as-received crystal, then cleaved by mechanical exfoliation with scotch tape, which removed the top-most layers of MoS_2, leaving a freshly exposed crystal surface. The latter was used as the working electrode and platinum foil as counter electrode, both of them being connected to a DC power supply (Agilent 6614C), using crocodile clips, and fixed at a distance of ~2 cm from each other in the solution. In this configuration, about two-thirds of the MoS_2 electrode was immersed in the electrolyte. In the first step, 20 mL of an aqueous 4 M KCl solution was used as the electrolyte and a negative voltage of -20 V was applied to the MoS_2 electrode for 30 min, to promote its delamination. Then, functionalization was carried out by substituting the electrolytic medium by 20 mL of iodoacetic acid solution and a negative voltage of -5 V was applied for 1 h. Although different solvents (ethanol, isopropanol, water and supporting electrolytes for iodoacetic acid were tested, the functionalization was considered to be optimal using an aqueous solution of 0.05 M iodoacetic acid and 0.15 M Na_2SO_4. After the electrochemical treatment, the most expanded part of the cathode, which roughly corresponded to the lower half of the MoS_2 piece, was recovered, thoroughly washed with water, dried overnight under reduced pressure at room temperature and finally stored in glass vials for subsequent use.

1.3 *Post-processing of the functionalized products: nanosheet extraction and dispersion in water*

To obtain individualized MoS_2 nanosheets (NSs) from the electrochemically treated material, the latter was first cut into smaller pieces with the aid of a scalpel and then transferred to water at nominal concentration of 2 mg mL^{-1}, where it was bath-sonicated (J.P. Selecta Ultrasons system, 40 kHz) for 3 h. After standing undisturbed for 24 h to allow sedimentation of the poorly exfoliated fraction of the material, the upper ~75% of the supernatant volume was collected from the sonicated dispersion and kept for subsequent use.

1.4 *Characterization techniques*

The material was characterized by UV-vis absorption spectroscopy, zeta-potential measurements, atomic force microscopy (AFM), field-emission scanning electron microscopy (FE-SEM), Raman spectroscopy, X-ray photoelectron spectroscopy (XPS), attenuated total reflection Fourier transform infrared (ATR-FTIR) spectroscopy, electron paramagnetic resonance (EPR) spectroscopy as well as by measurement of its water contact angle. UV-vis absorption (extinction) spectra were obtained for colloidal dispersions of the exfoliated MoS_2 material in a double-beam Heλios α spectrophotometer (Thermo Spectronic) using quartz cuvettes with an optical path length of 1 cm. The average lateral size and thickness of the dispersed NSs along with their concentration in the supernatant suspension were estimated by means of UV-vis absorption (extinction) spectroscopy using the metrics developed by Backes *et al* for this 2D material [1]. Zeta-potential measurements were carried out at a temperature of 25 ºC with a Malvern Zetasizer Nano ZSP apparatus from Malvern Instruments Ltd. using ~1 mL of MoS_2 dispersion at a concentration of 0.02 mg mL^{-1}. AFM measurements were carried out with a Nanoscope IIIa Multimode apparatus (Veeco Instruments) in the tapping mode of operation, using silicon cantilevers with nominal spring constant and resonance frequency of ~40 N m^{-1} and 250–300 kHz, respectively. Samples for AFM were prepared by drop-casting a small volume (~10 μL) of a low-concentration MoS_2 NS dispersion in isopropanol (~0.03 mg mL^{-1}) onto highly oriented pyrolytic graphite and allowing it to dry under ambient conditions first and then, under vacuum at room temperature. FE-SEM images were recorded on a Quanta FEG

apparatus (FEI Company) operated at 25 kV. Raman spectra were recorded on a Horiba Jobin-Yvon LabRam instrument at a laser excitation wavelength of 532 nm (green line). A low incident laser power (~0.2 mW) was employed to avoid damage to the NSs. XPS was accomplished on a SPECS system, working at a pressure below 10^{-7} Pa with a monochromatic Al K_{α} X-ray source (1486.7 eV) operated at 14.00 kV and 150 W. For Raman spectroscopy, XPS and contact angle measurement, specimens were prepared in the form of thin films by drop-casting MoS_2 NS dispersions (in isopropanol, in the case of the nonfunctionalized NSs and in water, in the case of the functionalized ones) onto stainless steel discs, which were allowed to dry at room temperature. ATR-FTIR spectra were registered on a Nicolet 8700 spectrometer (Thermo Scientific) with a diamond ATR crystal. Specimens for ATR-FTIR spectroscopy were prepared in the form of freestanding, paper-like films by vacuum-filtering aqueous dispersions of the functionalized MoS_2 material through silver membrane filters. EPR spectra were recorded with a X-Band (9.4 GHz) Bruker ELEXSYS E500 spectrometer using a magnetic field modulation amplitude of 1 G, a modulation frequency of 100 kHz and a microwave power of ~20 mW. Contact angles were measured by dropping 2 μL of water on the films with a pipette and immediately taking images of the droplet with a standard digital camera with an attached macro lens. Analysis of the recorded images with ImageJ software was carried out to determine the value of the contact angle.

1.5 Catalytic tests

For the catalytic tests, aqueous aliquots (2.5 mL) containing MoS_2 catalyst at concentration of 7 $\mu g\ mL^{-1}$ and the substrate molecule (a nitroarene or an organic dye at a concentration of 0.12 or 0.06 mM, respectively) as well as a large excess of $NaBH_4$ (72–110 mM) were prepared in quartz cuvettes with an optical path length of 1 cm. Immediately after preparation, the cuvettes were transferred to an UV-vis absorption spectrophotometer and the reaction progress was monitored by recording the corresponding kinetic profiles. The latter were obtained by measuring the temporal evolution of absorbance at the wavelength of a characteristic peak of the substrate molecule, namely, at 400, 416, 380, 410, 270, 461 and 675 nm for 4-NP, 2-NP, 4-NA, 2-NA, NB, MB and MO, respectively. To check the performance of the catalyst at higher concentrations of substrate, 1.2 mM and 12 mM 4-NP aqueous solutions were

reduced by $NaBH_4$ (1.1 M) in the presence of 7 μg mL^{-1} MoS_2 catalyst. To record the kinetic profiles, the absorbance of the catalytic medium was directly measured, without dilution, monitoring a wavelength different to that of the absorbance maximum (400 nm) to avoid saturation of the signal. Specifically, a wavelength of 455 nm was used for 1.2 mM 4-NP and 479 nm for 12 mM 4-NP. To test the catalyst for the reduction of mixtures of nitroarenes, a binary mixture of 0.12 mM 4-NA and 0.24 mM 2-NA and a ternary mixture of 0.06 mM 4-NP, 0.06 mM 4-NA and 0.12 mM 2-NA were reduced by $NaBH_4$ in the presence of MoS_2 catalyst at a concentration of 3.5 μg mL^{-1}. The kinetic profiles of the mixtures were followed by monitoring the absorbance at a wavelength of 400 nm. For every kinetic profile, a blank experiment, i. e., a kinetic run were no catalyst was added to the reaction medium, was recorded. To facilitate their recovery and re-use, the functionalized MoS_2 NSs were immobilized onto commercial polyurethane foam. To this end, the foam was first cut into cylinders ~1 cm in diameter and ~1 cm in height and cleaned by bath-sonication during 20 min in ethanol. After drying under vacuum, the polyurethane foam cylinders were soaked into ~10 mg L^{-1} aqueous MoS_2 dispersion and then dried at room temperature under reduced pressure. To increase the amount of loaded NSs, the soaking/drying process was carried out several times (typically three), after which the original white color of the polyurethane foam turned into an olive green tone. For the catalytic tests, the MoS_2-loaded foam cylinders were immersed into the substrate/$NaBH_4$ reaction mixture. After reaction completion, the cylinders were removed from the solution, rinsed with water and allowed to dry before being used in the next catalytic cycle.

2 Additional characterization of the functionalized MoS_2 material

2.1 Additional XPS spectra of MoS_2 nanosheets functionalized with iodoacetic acid

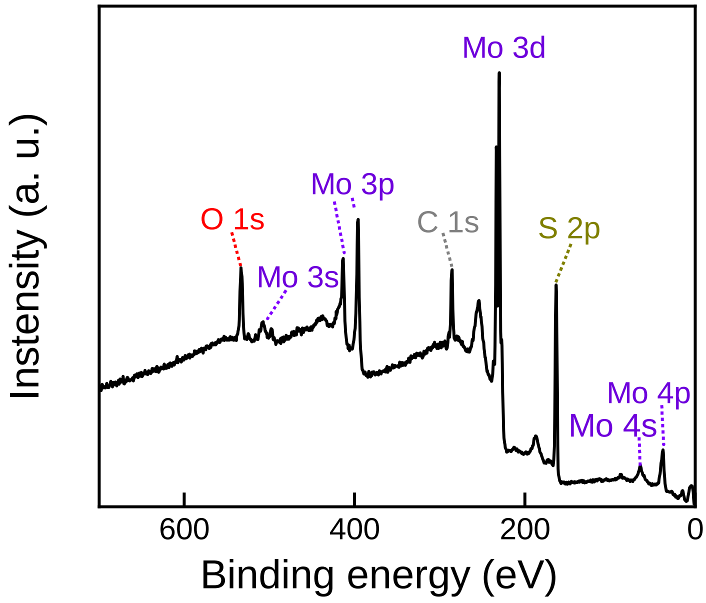

Figure S1. XPS survey spectrum of cathodically functionalized MoS_2 nanosheets. The main bands are labeled for clarity. The fact that no signal was detected in the 635–615 eV binding energy range, which is characteristic of the main XPS core level of iodine (I 3d) confirmed that this element was absent from the material.

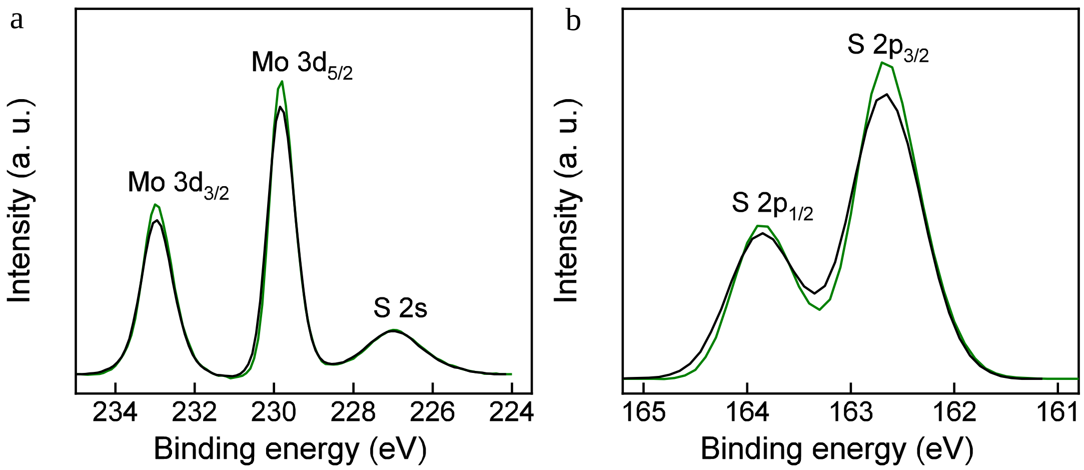

Figure S2. Background-subtracted, high resolution XPS **(a)** Mo 3d and **(b)** S 2p core level spectra of the nonfunctionalized (black trace) and functionalized (green trace) MoS_2 nanosheets. The symmetrical shape and position of the $3d_{5/2}$ and $3d_{3/2}$ components

of the Mo 3d doublet band (~229.8 and 233 eV, respectively) and of the $2p_{3/2}$ and $2p_{1/2}$ components of the S 2p doublet band (~162.7 and 163.9 eV, respectively) was consistent with 2H phase-only materials. A downshift of ~0.8 eV in the position of all these components should be expected for the 1T´ phase.

2.2 Estimation from the degree of functionalization from XPS data

2.2.1 <u>Photoelectron emission cross-section of MoS_2 for electrons from S 2p core level as a function of emission depth</u>

When photo-emitted electrons pass through a layer of material with thickness *d*, the intensity of the corresponding signal, I_0, is exponentially attenuated according to the following equation, often referred to as the Beer–Lambert law or the straight-line approximation:

$$I=I_0 exp(-d/Lcos\ \theta) \quad (1)$$

where θ is the angle of emission of the electrons from the surface normal and *L* is the attenuation length, which gathers both elastic and inelastic scattering effects. By considering electrons that emerge at θ=90 º to the sample surface, which was the configuration used for the XPS measurements reported here, the equation simplifies to:

$$I=I_0 exp(-d/L) \quad (2)$$

Incidentally, the approximate probe depth or information depth (ID) is defined as the sample thickness from which a specified percentage (e.g., 95%) of the detected signal originates. From eq. (2), some 95% of the signal will emanate from a depth lower than *3L* and thus:

$$ID=3L \quad (3)$$

In earlier approaches to estimate *L*, the inelastic mean free path (λ) was calculated first and then, in a simplistic way, *L* was considered to be 10 per cent less than λ [2]:

$L=0.9\lambda$ (4)

More recently, independent universal curves for λ [3] and *L* [4] have been derived, which display the lowest deviations from the experimental data reported to this day amongst the different available equations [including the well-known TPM-2M equation by Tanuma, Powell and Penn [5], which is offered as an option for the calculation of λ for inorganic compounds in the NIST electron inelastic mean free path database from predictive formulae [6]]. Specifically, the equation of the universal curve for *L* is:

$$L=\frac{(5.8+0.0041Z^{1.7}+0.088E^{0.93})a^{1.82}}{Z^{0.38}(1-W)}, \qquad (5)$$

Apart from the energy of the photo-emitted electrons *E*, the only parameters involved in the calculation of *L* are the average atomic number *Z* and the average atomic size *a* for the corresponding material. In addition, for strongly bonded materials, such as oxides and alkali halides, a small extra term is included for the heat of formation *H* (where $W=0.06H$). In the case of 2H-MoS_2, H=276.14 KJ mol^{-1}.

The simplest approach to estimate *Z* for an inorganic compound with chemical formula G_gH_h is to calculate an average *Z* from the molecular formula as follows:

$$Z=\frac{gZ_g+hZ_h}{g+h} \qquad (6)$$

In the case of MoS_2, *Z* ~24.67 Da.

The atomic size *a* is deduced from the relation:

$$a^3=\frac{10^{21}M}{\rho N_A\,(g+h)}\,(nm^3) \qquad (7)$$

where ρ is the density in g cm^{-3}, N_A is Avogadro's number, and *g* and *h* are the stoichiometric coefficients in the molecular formula G_gH_h of a molecule with molecular mass *M*. In the case of 2H-MoS_2, using M=160.07 Da and ρ=5.03 g cm^{-3} yields a~0.26 nm.

The kinetic energy of the electrons photo-emitted from the S 2p core level using an Al K_α X-ray source is 1326.6 eV. Substituting *E* by the latter value and the aforementioned

values of H, Z and a for 2H-MoS_2 in the expression for L (eq. 5) yields a value of ~2.1 nm (probe depth of ~6.3 nm by eq. 3). We note that a similar value for L (~2.2 nm) is obtained by first calculating λ by the TPM-2M equation (using the values for M and ρ specified above, number of valence electrons of 18 and a band gap of 1.2 eV), and then L through eq. 4.

2.2.2 Contribution of the outermost sulfur atoms to the total sulfur signal

Once L for the S 2p sulfur signal is calculated, eq. 2 can be used to estimate how much the outermost sulfur atoms contribute to the total sulfur signal. As mentioned above, only electrons that emerge at θ=90 º to the sample surface are considered. The surface of the 2H-MoS_2 crystal will be constituted by its topmost (0001) basal plane, the Z axis of the crystalline structure being parallel to the photoemission. An ideal 2H-MoS_2 crystal shows sulfur planes every 3.186 Å (intra trilayer, i.e., distance between sulfur planes in a S-Mo-S sandwich) and every 2.959 Å (inter trilayer, i. e., distance between sulfur planes of adjacent S-Mo-S sandwiches) along its Z axis. To calculate the contribution of each sulfur plane to the total intensity of photo-emitted S 2p electrons, eq. 2 was used to calculate the percentage of out-coming, nonattenuated signal at the corresponding depth of each plane, except for the topmost sulfur plane, for which no attenuation was considered. The ratio of the contribution of the outer plane to that from all the sulfur planes which contribute to the signal amounts to ~14 %.

2.2.3 C1s core level XPS spectrum of bulk MoS_2 crystal functionalized with iodoacetic acid

If we consider, as explained in the main text, that all the acetic groups are grafted to the outermost sulfur atoms, then the ratio of the signal corresponding to COOH (component at ~289 eV in the C 1s core level band depicted in Fig. S3 below these lines) to that corresponding to topmost sulfur plane can be taken as an indication of the extent of functionalization. This yields a value of ~0.10 molecular groups inserted per surface sulfur atom.

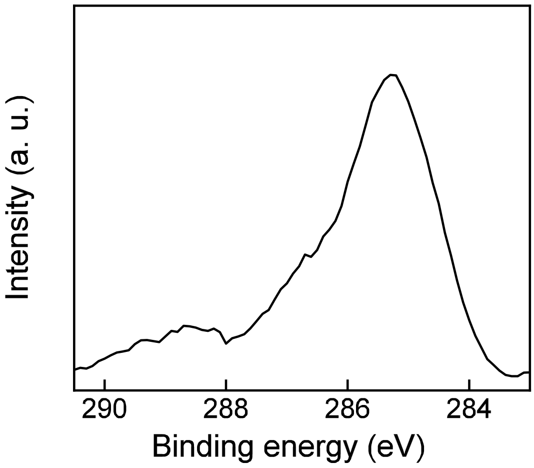

Figure S3. Background-subtracted, high resolution XPS C 1s core level spectrum of bulk MoS_2 crystal functionalized through cathodic treatment with iodoacetic acid.

2.3 XPS spectra of MoS_2 nanosheets functionalized with iodoaniline

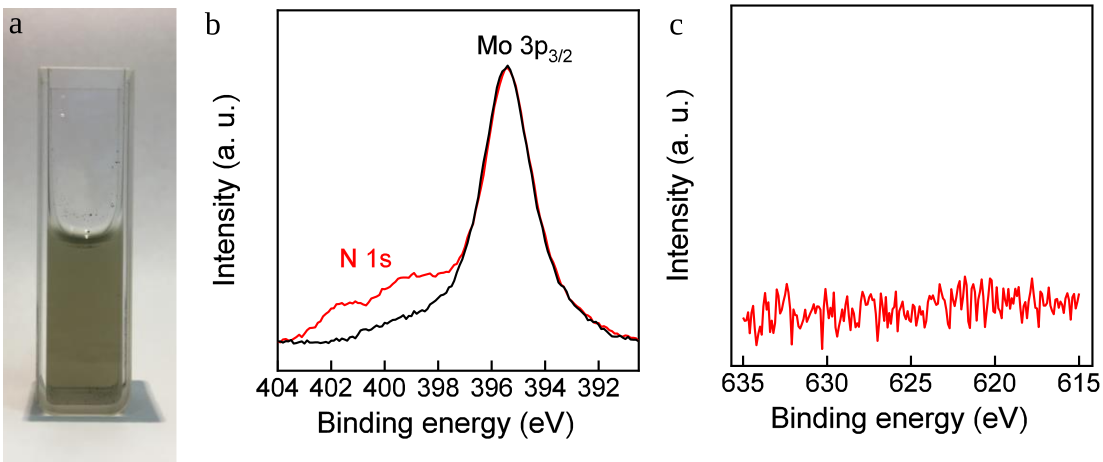

Figure S4. MoS_2 nanosheets functionalized with iodoaniline (a) Digital photograph of the aqueous dispersion obtained by sonication in water of cathodically expanded MoS_2 with a subsequent electrochemical treatment at -5 V with 0.25 M iodoaniline in ethanol. **(b)** Background-subtracted, normalized high resolution XPS spectra of the nonfunctionalized MoS_2 NSs (black trace) and MoS_2 functionalized with iodoaniline (red trace) in the binding energy range from 404 to 391.5 eV. In this range, only the main component of the Mo 3p doublet band, Mo $3p_{3/2}$, which is symmetrical and centered at 395.4 eV, was detected for the nonfunctionalized NSs (black trace). However, the functionalized material displayed two additional bands which correspond to nitrogen in aniline moieties. Specifically, the two N 1s components detected at

~399.4 and ~401.7 eV were assigned to amine in neutral and positively charged form, respectively [7]. **(c)** I 3d binding energy range for the MoS_2 nanosheets functionalized with iodoaniline. The absence of the main band of iodine in the MoS_2 NSs treated with iodoaniline that any un-reacted iodoaniline had been effectively removed from the surface by the purification process and thus confirmed that the detected aniline groups were grafted on MoS_2 surface, instead of just adsorbed on it.

2.4 Functionalization using a reducing agent as an electron source

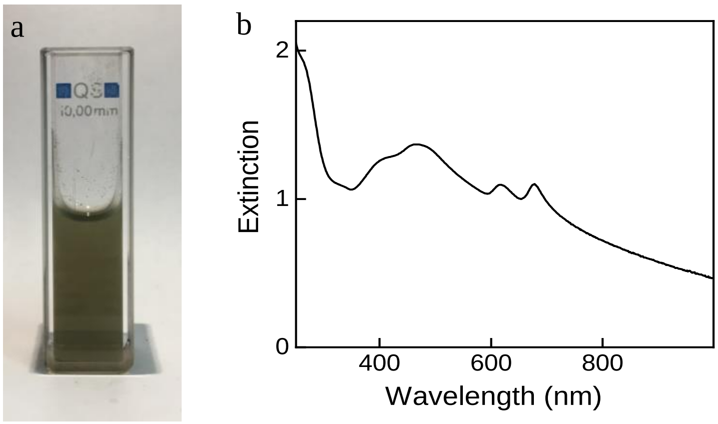

Figure S5. (a) Digital photograph and **(b)** UV-vis extinction spectrum for the dispersion obtained by sonication during 1 h of a cathodically expanded MoS_2 crystal in an aqueous solution of 0.25 M iodoacetic acid and 0.25 M sodium borohydride.

2.5 Presence of bismuth in some of the MoS_2 materials

It has been recently reported that geological MoS_2 crystals, such as the starting material in this work, contain bismuth impurities in concentrations high enough to be detected by XPS [8]. Specifically, the bismuth impurity has been found to appear mainly as the most thermodynamically stable form of bismuth oxide, Bi_2O_3. In the present work, the presence of micron-size clusters of bismuth in the MoS_2 starting material was indeed confirmed by EDX. Correspondingly, bismuth was detected in some of the samples analyzed by XPS. Bi^{3+} $4f_{5/2}$ spin-orbit component appears in a similar range to that where the S 2p component for S-C bond is expected to appear (~163-164 eV) [9,10]. Thus, special care was taken not to mistake Bi^{3+} $4f_{5/2}$ for the S-C component in S 2p spectrum, by widening the acquired range for the S 2p spectrum downwards to include Bi^{3+} $4f_{7/2}$ as well, thus confirming the presence of bismuth.

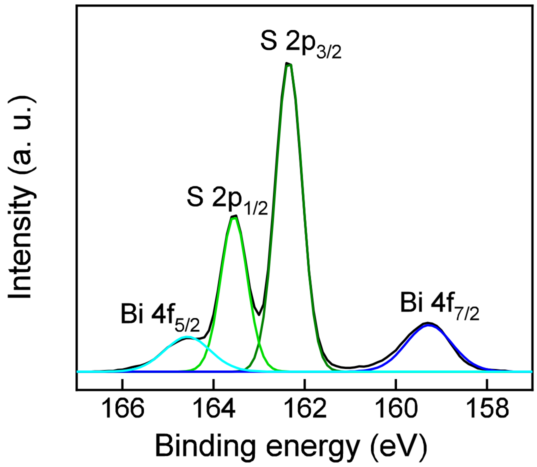

Figure S6. Background-subtracted, high resolution XPS S 2p and Bi 4f core level spectra for one of the batches where bismuth was detected. As expected for an ideal sulfur signal, the S 2p spin–orbit doublet consists of S $2p_{1/2}$ (light green trace) and S$2p_{3/2}$ (dark green trace) components with a 1:2 peak area ratio between and spin-orbit splitting constant of 1.2 eV, The position of the of S $2p_{1/2}$ and S$2p_{3/2}$ components of the S 2p doublet band (~163.9 and 162.7 eV, respectively) as well as their full width at half maximum (FWHM) of just 0.7 eV was consistent with a 2H phase-only material. The Bi 4f doublet displayed Bi $4f_{5/2}$ and Bi $4f_{7/2}$ components split apart by 5.3 eV and in 3:4 area ratio, as expected, which were centered at binding energy of 164.3 eV and 159.3 eV, respectively, which corresponded to Bi(III).

2.6 Difference C 1s spectrum of functionalized and nonfunctionalized MoS_2

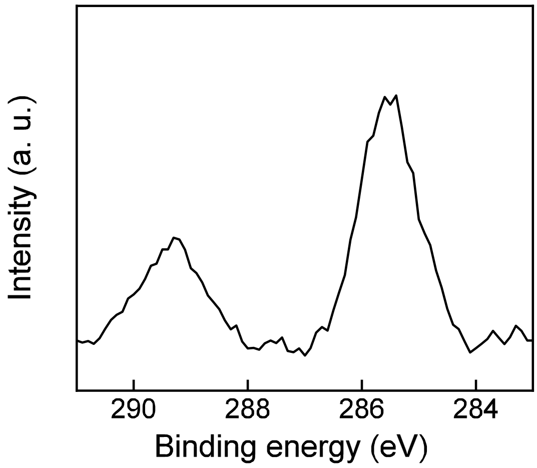

Figure S7. Difference C 1s XPS spectrum of the functionalized and the nonfunctionalized MoS_2 nanosheets (green and black traces in Figure 2g of the main text, respectively).

2.7 ATR-FTIR spectrum of functionalized MoS_2

The ATR-FTIR spectrum of functionalized MoS_2 exhibited some additional indication of functionalization.

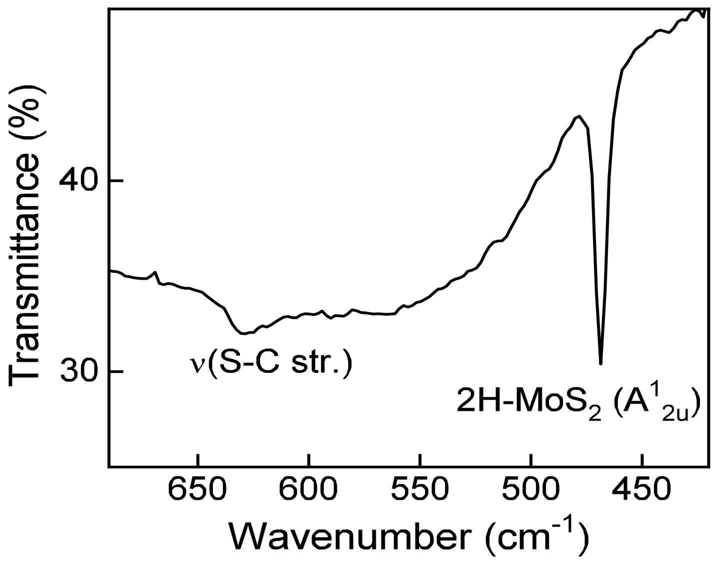

Figure S8. ATR-FTIR spectrum of the functionalized MoS_2 NSs.

Specifically, a weak band located at ~630 cm^{-1} can be ascribed to S−C stretching vibrations [9,11] arising from acetic acid groups grafted onto the NSs via sulfur atoms. This band was not very intense even in the case of functionalized 1T-phase MoS_2 monolayers, where the degree of derivatization with iodoacetic acid was ~20 percent [10]. Thus, it is expected to be weak for the current materials, where, as explained in the main text, the multi-layered nature of the MoS_2 NSs implies that the percentage of (surface) sulfur atoms that are covalently bonded to carbon must be very small.

The sharp absorption peak at ~470 cm^{-1} is due to the A^1_{2u} phonon of 2H-MoS_2, which involves asymmetric translation of both Mo and S atoms parallel to the c axis [12]. The bands associated to the acetic group were also observed (range not shown) but they

would be compatible with just adsorption (noncovalent fuctionalization) of iodoacetic acid.

2.8 EPR spectrum of functionalized MoS_2

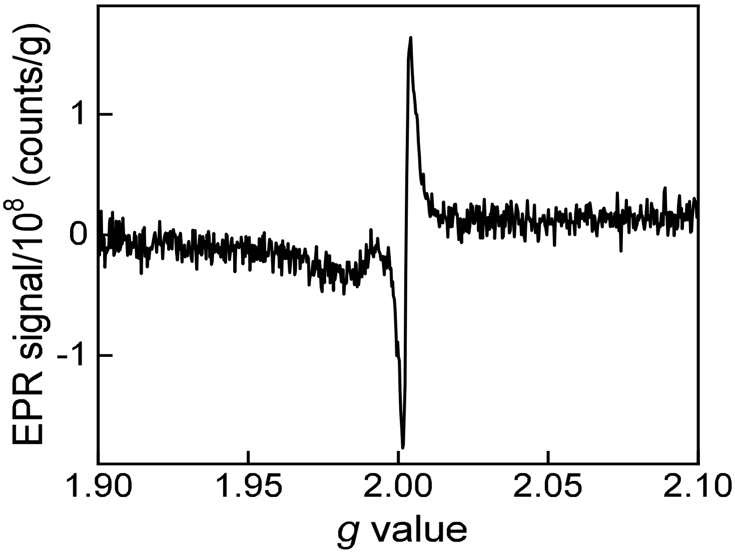

Figure S9. Representative first-derivative X-band EPR spectrum of the functionalized MoS_2 NSs. The unsaturated molybdenum atoms in edges and sulfur vacancies give rise to an EPR signal with g~2 [13,14].

3 Additional information on the performance of functionalized MoS_2 nanosheets as catalysts

3.1 UV-vis spectra of reactants and products

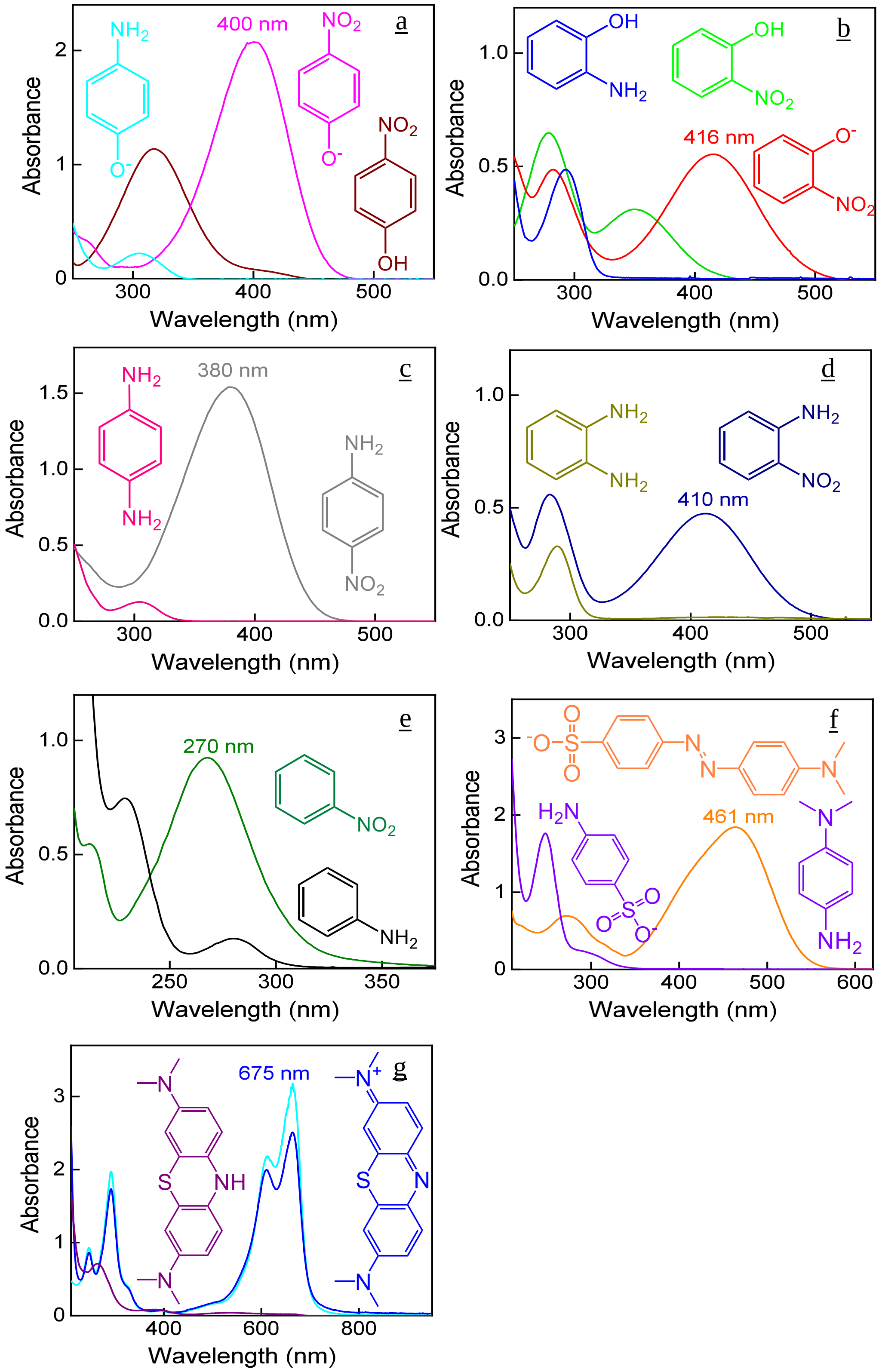

a
400 nm
Absorbance
Wavelength (nm)
b
416 nm
c
380 nm
d
410 nm
e
270 nm
f
461 nm
g
675 nm

Figure S10. Chemical structure and UV-vis spectra of the reactants and products involved in the different reduction reactions where functionalized MoS_2 NSs have been tested as catalyst: (a) 4-NP (brown trace), 4-nitrophenolate (pink trace) and 4-aminophenol (cyan trace); **(b)** 2-NP (green trace), 2-nitrophenolate (red trace) and 2-aminophenol (blue trace); **(c)** 4-NA (gray trace) and its reduction product, *p*-phenylendiamine (fuchsia trace); **(d)** 2-NA (navy trace) and its reduction product, *o*-phenylendiamine (dark yellow trace); **(e)** nitrobenzene (olive trace) and aniline (black trace); **(f)** MO (orange trace), which is first reduced to the corresponding azo product (not shown) [15], which is followed by -HN-NH- bond dissociation to yield N,N-dimethyl-benzene-1,4-diamine and 4-amino-benzenesulfonate (both shown in violet trace) [16]; **(g)** MB (cyan trace), MB in the basic medium provided by $NaBH_4$ (blue trace) and its reduction product, leucomethylene blue (purple trace) [17,18]. The wavelength chosen to follow the kinetics of the corresponding reactions, which was in all cases the location of an absorption maximum of the reactant, is indicated for clarity.

3.2 Extinction spectrum of the catalyst

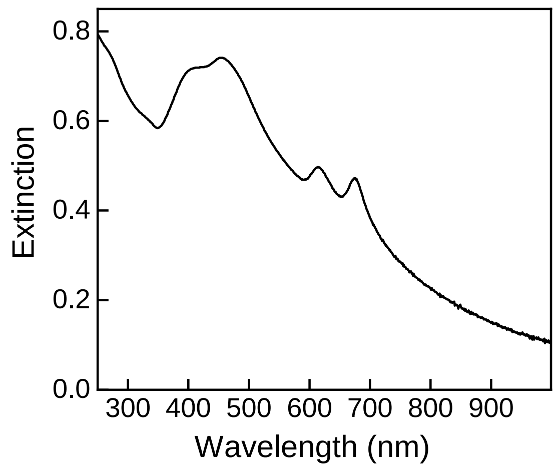

Figure S11. Extinction spectrum of the catalyst at a concentration of 7 mg L^{-1}. The extinction value for the catalyst at the wavelengths monitored for the reduction reactions of the different substrates was ~0.6 for all the substrates except for MB, for which it was 0.4 (as expected from the extinction value at λ_{max}~400 and ~675 nm, respectively).

3.3 Comparison of the catalytic activities of functionalized MoS_2 nanosheets with those reported in the literature for other MoS_2- and non-noble metal-based catalysts

Table S1. Catalytic activity (defined as number of moles of reactant converted per mole of catalyst used per unit time) of the functionalized MoS_2 NSs towards the reduction of 4-nitrophenol compared with that of different types of MoS_2 nanostructures, graphene-derived materials and non-noble metal-based catalysts reported in the literature.

**Catalytic system**	**Catalytic activity (h^{-1})**	**Ref.**
Functionalized-2H MoS_2 NSs	63	Present work
S vacancy-decorated, ultrasound-exfoliated 2H MoS_2 NSs	6.6	19
Li-exfoliated 2H-phase MoS_2 NSs	0.9	20
Li-exfoliated 1T-phase MoS_2 NSs	44.4	20
Hydrothermally synthesized 2H MoS_2 NSs supported onto Fe_3O_4 particles	2.4	21
GMP-stabilized sonicated 2H MoS_2 NSs	2.6–7.8	22
GMP-stabilized cathodically exfoliated 2H MoS_2 NSs	21.4	23
N-doped RGO foam	0.07	24
RGO NSs capped with poly(diallyldimethylammonium chloride)	0.10	25
Hydrothermally synthesized 2H MoS_2 NSs	5.4	26
Hydrothermally synthesized MoS_2 NSs intercalated in pillared montomorillonite	24	26
Hydrothermally synthesized 1T MoS_2 NSs	21	27
Bi_2Te_3-hydrothermally synthesized 1T MoS_2 heterostructure	67.2–100.8	27
Hydrogel network with embedded Co nanoparticles (NPs)	16.2	28

Ni nanoparticles on silica nanotubes	1.8	29
Hybrid Ni nanoparticles/N doping carbon on diatomite	0.85	30
Cu NPs on carbon microspheres	0.2	31
Cu and Sn sponges/dendrites	1.2–1.8	32
Co particles-decorated carbon microspheres	0.06–0.24	33
Co nanocrystals on reduced graphene oxide (RGO)	0.6	34
Ni NPs supported onto carbon black	26.4	35
Nanostructured zero-valent iron	78	36
Graphene stabilized CuNi nanocomposite	12	37
MOF-derived Ni based N-doped mesoporous carbon	2.4	38
Co NPs embedded in hierarchically porous N-doped carbon frameworks	18	39
Hexagonal Ni plates on RGO	0.44	40
Hollow porous Cu particles from silica-encapsulated Cu_2O nanoparticle aggregates	3	41
Co@BN core–shell nanoparticles	0.38	42
Co NPs embedded into ordered mesoporous carbon	7.8	43
NiO hollow nanospheres	4.2	44
Ultrafine Cu_2O nanoparticles on cubic mesoporous carbon	9.6	45
Cu NPs on oxidized boron nitride	27	46
Cu NPs immobilized by layered Ti_3C_2 MXene	51	47
Cu and Co NPs doped N-containing carbon frameworks	63	48

Table S2. Catalytic activity of the functionalized MoS_2 NSs towards the reduction of other nitroarenes and organic dyes compared with that of different types of MoS_2 nanostructures, and non-noble metal-based catalysts reported in the literature.

Catalytic system	Catalytic activity (h^{-1})	Ref.
**2-nitrophenol**		
Acetic acid-functionalized, cathodically exfoliated 2H MoS_2 NSs	340	Present work
N/O-doped, hydrothermnally synthesized MoS_2 NSs	0.3	49
Li-exfoliated 1T MoS_2 NS-RGO hybrid paper	3 (*)	50
NiO NSs	0.2	49
$NiMoO_4$ NPs	0.3	51
$CuFe_2O_4$ NPs	133	52
$NiFe_2O_4$ NPs	17	52
CuO NPs supported on γ-Al_2O_3	0.5	53
Carbon black-supported Ni NPs	27	54
$Fe_2(MoO_4)_3$ NPs	7	55
RGO-supported Ni NPs	176 (*)	56
Fe@SiO_2-supported Ni NPs	60.84	57
Poly(methacrylic acid)-supported Cu NPs	217	58
**4-nitroaniline**		
Acetic acid-functionalized, cathodically exfoliated 2H MoS_2 NSs	180	Present work
S vacancy-decorated, ultrasound-exfoliated 2H MoS_2 NSs	43	19
Li-exfoliated 1T MoS_2 NSs	83.4	20
GMP-stabilized, cathodically exfoliated 2H MoS_2 NSs	140	23
Bi_2Te_3-hydrothermally synthesized 1T MoS_2 heterostructure	106.8	27
Li-exfoliated 1T MoS_2 NS-RGO hybrid paper	6	50

Carbon-embedded Cu NPs	1	59
RGO-supported Ni NPs	0.1	60
MnO_2 NSs	0.7	61
Fe_3O_4 NPs	0.9	61
Fe@SiO_2-supported Ni NPs	179.8	57
Mesoporous polyaniline-supported Cu NPs	84	62
Pectin-capped Cu NPs	90	63
Poly(methacrylic acid)-supported Cu NPs	54	58
Fe_3O_4@Cu NPs	21	64
**2-nitroaniline**		
Acetic acid-functionalized, cathodically exfoliated 2H MoS_2 NSs	90	Present work
Li-exfoliated 1T MoS_2 NS-RGO hybrid paper	6 (*)	50
$Bi_2O_2CO_3$/$CoFe_2O_4$ hybrid nanostructures	0.1	65
DNA-supported Ni NPs	17	66
RGO-supported Ni NPs	44(*)	56
Fe@SiO_2-supported Ni NPs	146.7	57
Pectin-capped Cu NPs	22.5	63
**nitrobenzene**		
Acetic acid-functionalized, cathodically exfoliated 2H MoS_2 NSs	78	Present work
S vacancy-decorated, ultrasound-exfoliated 2H MoS_2 NSs	11	19
Li-exfoliated 1T MoS_2 NS-RGO hybrid paper	7.8 (*)	50
Bi_2Te_3-hydrothermally synthesized 1T MoS_2 heterostructure	960 (**)	27
Ni NP-polyvinylamine/SBA-15 hybrid	1.3	67
Fe_3O_4/β-alanine-acrylamide-Ni nanocomposite	770	68
DNA-supported Ni NPs	25	66
RGO-supported Ni NPs	59 (*)	56
Mesoporous polyaniline-supported Cu NPs	4.2	62

**methyl orange**		
Acetic acid-functionalized, cathodically exfoliated 2H MoS_2 NSs	71	Present work
S vacancy-decorated, ultrasound-exfoliated 2H MoS_2 NSs	24	19
Li-exfoliated 1T MoS_2 NSs	150	20
Hydrothermally synthesized 2H MoS_2 NSs intercalated in pillared montmorillonite	13	69
Poly(methacrylic acid)-supported Cu NPs	35	58
Fe_3O_4@Cu NPs	10	64
Fe_3O_4-cellulose-Cu nanocomposite	0.9	70
**methylene blue**		
Acetic acid-functionalized, cathodically exfoliated 2H MoS_2 NSs	44	Present work
S vacancy-decorated, ultrasound-exfoliated 2H MoS_2 NSs	16	19
Fe_3O_4@Cu NPs	86	64
Spherical montmorillonite-supported MoS_2 NSs	70	71
Solvothermally synthesized, amorphous MoS_2 NPs	0.01	72
α-MnO_2 nanorods	0.2	73
Cu NPs supported onto Si nanowire array	66	74

(*) in 1:1 water/methanol solvent.
(**) in methanol solvent

3.4 Reaction mechanism

The mechanism of nitroarene and dye reduction catalyzed by the present functionalized MoS_2 is expected to be the same that we previously proposed for vacancy-decorated 2H-MoS_2 [19], i. e., a mechanism involving the catalytically active sites in the NSs, such as sulfur vacancies and edges, which possess local metallic character. The fact that the functionalization takes place near an active site of the MoS_2 NSs does not promote the deactivation or blocking of the latter. As the acetic moiety is bonded to the NSs via a sulfur atom near a sulfur vacancy, the sulfur vacancy remains "vacant", and it is thus

preserved and active. In contrast, a usual passivation method would involve filling of the vacancy with a sulfur atom provided by an external source, e.g., through chemical reaction with alkanethiols [75–77]. Indeed, the EPR signal of the functionalized MoS_2 NSs (Fig. S9) is indicative of the presence of Mo-S dangling bonds. The presence of the negatively charged acetic moiety near the active site does affect the process in some way, as discussed below and in the main text, but the reaction mechanism remains basically the same.

When nanoparticles (NPs) of certain noble metals such as gold are used as catalysts for the catalytic reduction of nitroarenes with $NaBH_4$, the BH_4^- anion readily oxidizes, injecting electrons to the NPs, which thereby become a reservoir of excess electrons [78]. Upon adsorption of the substrate molecules onto the NPs, the excess electrons trigger their reduction. In such case, the catalytic reaction is insensitive to the pH of the aqueous medium [79]. In contrast, when metallic 1T-MoS_2 NSs are used as catalyst for the same type of reaction, the reduction does not occur at pH values above ∼11 [80], which is the pH range where the spontaneous hydrolysis of BH_4^- anion becomes arrested [81,82]. This implies that, in the latter case, some product of the hydrolysis of the BH_4^- anion, rather than BH_4^- anion itself, is involved in the reduction reaction. As the active sites in 2H-MoS_2 (sulfur vacancies and edges) possess a locally metallic character, the same mechanism proposed for 1T-MoS_2 applies for 2H-MoS_2, as has been indeed reported before for vacancy-decorated 2H-MoS_2 [19].

In basic solution below pH~11 the BH_4^- anion hydrolyzes spontaneously to give molecular hydrogen and BH_3OH^- species as reaction products.

$BH_4^- + H_2O \rightarrow BH_3OH^- + H_2$ (1)

H_2 molecule undergoes dissociative adsorption at sulfur vacancies in MoS_2 [83,84]. Although the resulting adsorbed H atoms could in principle react with a nitroarene/dye molecule to trigger its reduction, such a reduction reaction is inhibited by the fast recombination of the adsorbed H atoms into H_2 molecules, which is a very favorable process at the catalytic sites of MoS_2 (i.e., the Tafel step of hydrogen evolution reaction, HER) [85,86]. Indeed, previously reported control experiments in which a H_2 gas flow several orders of magnitude larger than the hydrolysis rate of BH_4^- was passed through a nitroarene solution in the presence of MoS_2 NSs showed that no reduction of the substrate took place [20].

In addition to H_2, the spontaneous hydrolysis of $NaBH_4$ yields the BH_3OH^- anion as a rather stable intermediate. In the basic medium of the reaction, the BH_3OH^- species is oxidized at a sulfur vacancy site on the MoS_2 surface with the assistance of hydroxide anions, to release the metaborate anion (BO_2^-), water, hydrogen and electrons [reaction (1) in Fig. S12] [87]:

$2BH_3OH^- + 6OH^- \rightarrow BO_2^- + 4H_2O + 3H_2 + 6e^-$ (2)

As previously reported control experiments where $NaBH_4$ was substituted by $NaBO_2$ in the reaction medium have shown, the generated BO_2^- is inactive as a reductant and thus will not participate in the catalytic reduction [20]. Molecular hydrogen is activated by the released electrons to give a negatively charged hydride (H^-) [88], which is very unstable, and thus would not form on the surface of defect-free MoS_2, but becomes stabilized when it adsorbs at sulfur vacancies by transferring excess electron charge to their neighboring unsaturated molybdenum atoms [89] [reaction (2)]. The vacancy-anchored, negatively charged hydride should readily trigger the hydrogenation of the nitro group in the nitroarene molecules through the well-known three-step sequence [36,90]; i.e., the nitro group is first converted to the nitroso group [reaction (3)], which is then reduced to a hydroxylamine species [reaction (4)] and finally to the amino group [reaction (5)]. In the case of the dyes, the generation of highly active hydride species at the sulfur vacancy sites by way of reactions (1) and (2) in Fig. S12 would subsequently trigger hydrogenation of the substrate.

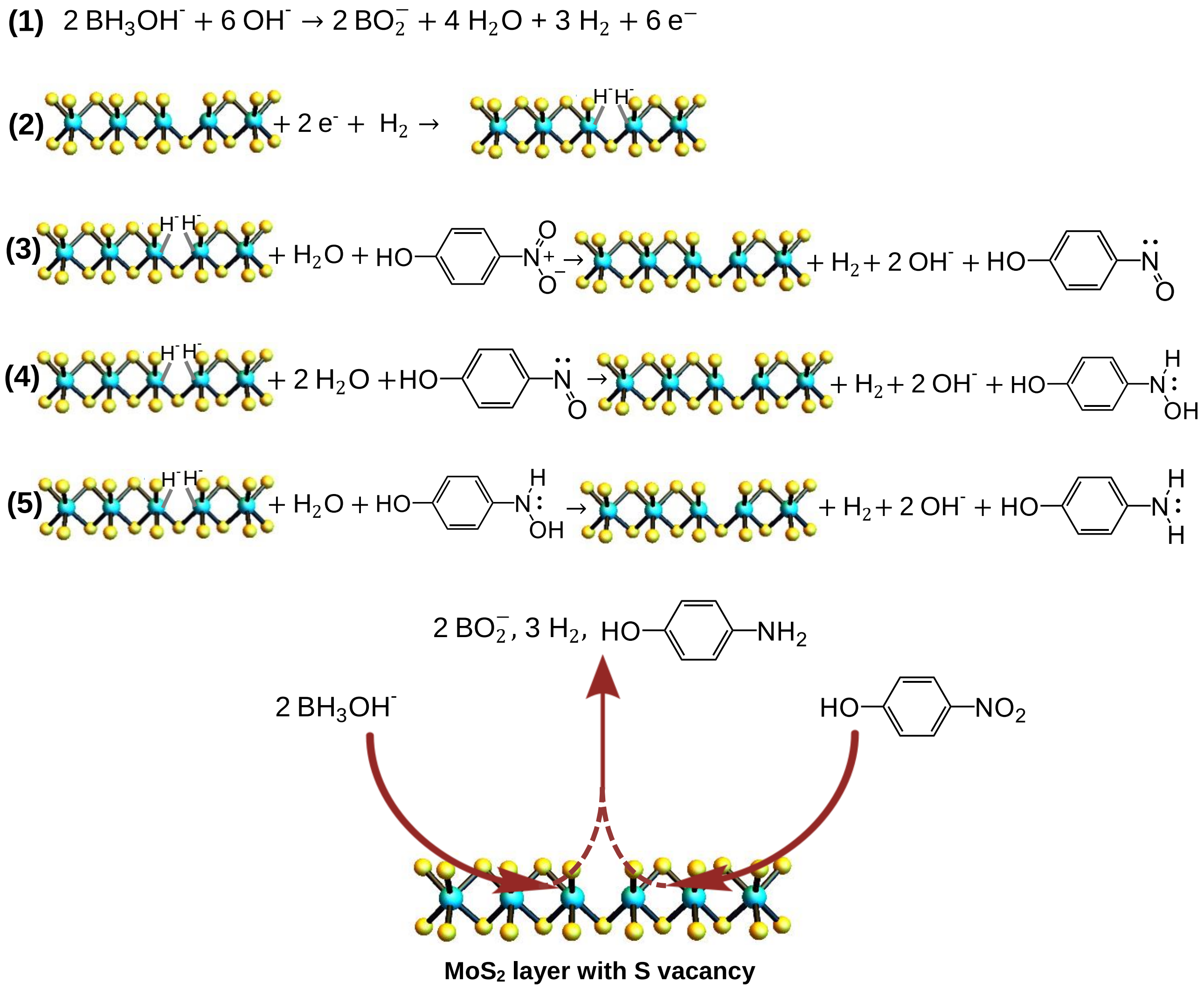

Figure S12. Proposed reaction mechanism for the reduction of 4-NP at the active sites of the MoS_2 nanosheets. Reaction (1): BH_3OH^- anion (the hydrolysis product of $NaBH_4$) is oxidized at a sulfur vacancy site on the MoS_2 surface with the assistance of hydroxide anions from the basic medium of the reaction, to release metaborate anion (BO_2^-), water, hydrogen and electrons. Reaction (2): the generated hydrogen is activated by the released electrons to give a negatively charged hydride (H^-), which adsorbs at the sulfur vacancy. The negatively charged hydride triggers the hydrogenation of the nitro group in the 4-NP molecule through the following three-step sequence: the nitro group is first converted to the nitroso group [reaction (3)], which is then reduced to a hydroxylamine species [reaction (4)] and finally to the amino group [reaction (5)]. The overall reaction is illustrated at the bottom.

As aforementioned, according to the proposed mechanism, the catalytic activity of a MoS_2 NS relies on the availability of stable adsorption sites for the H^- species, that is, unsaturated molybdenum atoms in MoS_2 edges and sulfur vacancies. Thus, the colloidal stability of the MoS_2 catalyst will have a major influence on the catalytic activity, as the adsorption sites for the H^- species in the MoS_2 layers will only be available as long as their surface remains exposed to the aqueous medium where catalysis takes place. If the layers aggregate, catalytic activity will consequently decrease.

The fact that the current materials perform better as catalysts in aqueous medium than previously reported surfactant-stabilized MoS_2 catalysts [19] could be partly related to the different way in which colloidal stabilization affects the catalytic sites in one and the other. In the present case, the covalently attached acetic group which imparts colloidal stability is grafted to a sulfur atom near the sulfur vacancy/edge, and thus the unsaturated molybdenum atoms remain available for adsorption of H^- at any time. However, in the surfactant-assisted stabilization, guanosine monophosphate (GMP) molecules adsorbed on the surface of MoS_2 by acid-base interaction between their nucleobase and some of the unsaturated molybdenum sites [22], thus blocking them. Hence, in the latter case, colloidal stability is attained at the expense of catalytic activity although not all catalytic centers will be blocked with GMP and, even those with GMP on, will be temporally available for catalysis as GMP is not irreversibly adsorbed. This was confirmed by the fact that the surfactant-stabilized MoS_2 NSs aggregated if many washing cycles were done, as washing led to their progressive removal from the NS surface and thus to a loss of colloidal stability. Thus, GMP will be desorbing and adsorbing from the surface of surfactant-stabilized MoS_2 NSs and unsaturated molybdenum sites will be available for catalysis when GMP eventually desorbs. We note that, in the case of the current functionalized MoS_2 NSs, the presence of the acetic group near the sulfur vacancy could introduce some steric impediment for the approaching substrate molecules. However, this effect will be minor in the case of diatomic vacancies, which are easily formed and stabilized [91].

References

[1] C. Backes, R.J. Smith, N. McEvoy, N.C. Berner, D. McCloskey, H.C. Nerl, A. O´Neill, P.J. King, T. Higgins, D. Hanlon, N. Scheuschner, J. Maultzsch, L. Houben, G.S. Duesberg, J.F. Donegan, V. Nicolosi, J.N. Coleman, Edge and confinement effects allow in situ measurement of size and thickness in liquid-exfoliated nanosheets, Nat. Commun. 5 (2014), 4576.

[2] J. F. Watts, J. Wolstenholme. An introduction to surface analysis by XPS and AES. Wiley, 2003.

[3] M. P. Seah. An accurate and simple universal curve for the energy-dependent electron inelastic mean free path. Surf. Interface Anal. 2012, 44, 497–533.

[4] M. P. Seah. Simple universal curve for the energy-dependent electron attenuation length for all materials. Surf. Interface Anal. 2012, 44, 1353–1359.

[5] S. Tanuma, C. J. Powell, D. R. Penn, Surf. Interface Anal. 1994, 21, 165.

[6] C. J. Powell, A. Jablonski, NIST Electron Inelastic-Mean-Free-Path Database – Version 1.2, National Institute of Standards and Technology, Gaithersburg, MD (2010). (available online at www.nist.gov/srd/nist71.cfm)

[7] E. Raymundo-Pinero, D. Cazorla-Amorós, A. Linares-Solano, J. Find, U. Wild, R. Schlögl, Structural characterization of N-containing activated carbon fibers prepared from a low softening point petroleum pitch and a melamine resin. Carbon 40 (2002) 597–608.

[8] M. G. Sales, L. Herweyer, E. Opila, S. McDonnell, MoS2 impurities: Chemical identification and spatial resolution of bismuth impurities in geological material. Appl. Surf. Sci. 2020, 508, 145256.

[9] D. Voiry, A. Goswami, R. Kappera, C.C.C. Silva, D. Kaplan, T. Fujita, M. Chen, T. Asefa, M. Chhowalla, Covalent functionalization of monolayered transition metal dichalcogenides by phase engineering, Nat. Chem. 7 (2015), 45-49.

[10] J.I. Paredes, J.M. Munuera, S. Villar-Rodil, L. Guardia, M. Ayán-Varela, A. Pagán, S.D. Aznar-Cervantes, J.L. Cenis, A. Martínez-Alonso, J.M.D. Tascón, Impact of covalent functionalization on the aqueous processability, catalytic activity, and

biocompatibility of chemically exfoliated MoS_2 nanosheets, ACS Appl. Mater. Interfaces 8 (2016), 27974-27986.

[11] G. Socrates. lnfrared Characteristic Group Frequencies: Tables and Charts,. Wiley: New York, NY, 1994, 2nd Ed.

[12] T. Livneh, J. E Spanier. A comprehensive multiphonon spectral analysis in MoS_2. 2D Mater, 2015, 2, 035003.

[13] G. Liu, H. Ma, I. Teixeira, Z. Sun, Q. Xia, X. Hong, S. C. E. Tsang, Hydrazine-assisted liquid exfoliation of MoS_2 for catalytic hydrodeoxygenation of 4-methylphenol. Chem. Eur. J. 2016, 22, 2910–2914.

[14] L. M. Martinez, C. Karthik, M. Kongara, S. R. Singamaneni, Paramagnetic defects in hydrothermally grown few-layered MoS_2 nanocrystals. J. Mater. Res. 2018, 33, 1565–1572.

[15] A. Furst, R. C. Berlo, S. Hooton, Hydrazine as a Reducing Agent for Organic Compounds (Catalytic Hydrazine Reductions). Chem. Rev. 1965, 65, 1, 51–68. DOI:10.1021/cr60233a00

[16] L.-Q. Zheng, X.-D. Yu, J.-J. Xu and H.-Y. Chen, Reversible catalysis for the reaction between methyl orange and $NaBH_4$ by silver nanoparticles. Chem. Commun., 2015, 51, 1050–1053.

[17] S. Pande, S. Jana, S. Basu, A. K. Sinha, A. Datta, T. Pal, Nanoparticle-Catalyzed Clock Reaction. J. Phys. Chem. C 2008, 112, 10, 3619-3626. DOI: 10.1021/jp7106999

[18] A. Mignani, S. Fazzini, B. Ballarin, E. Boanini, M. C. Cassani, C. Maccato, D. Barreca, D. Nanni. Mild fabrication of silica-silver nanocomposites as active platforms for environmental remediation, RSC Adv., 2015, 5, 9600–9606. DOI: 10.1039/C4RA14069A

[19] S. García-Dalí, J.I. Paredes, B. Caridad, S. Villar-Rodil, M. Díaz-González, C. Fernández-Sánchez, A. Adawy, A. Martínez-Alonso, J.M.D. Tascón, Activation of two-dimensional MoS_2 nanosheets by wet-chemical sulfur vacancy engineering for the catalytic reduction of nitroarenes and organic dyes, Appl. Mater. Today 20 (2020), 100678.

[20] L. Guardia, J. I. Paredes, J. M. Munuera, S. Villar-Rodil, M. Ayán-Varela, A. Martínez-Alonso and J. M. D. Tascón, Chemically exfoliated MoS_2 nanosheets as an efficient catalyst for reduction reactions in the aqueous phase, ACS Appl. Mater. Interfaces, 2014, 6, 21702–21710.
[21] T. Lin, J. Wang, L. Guo and F. Fu, Fe_3O_4@MoS_2 Core–shell composites: preparation, characterization, and catalytic application, J. Phys. Chem. C, 2015, 119, 13658–13664.
[22] M. Ayán-Varela, Ó. Pérez-Vidal, J. I. Paredes, J. M. Munuera, S. Villar-Rodil, M. Díaz-González, C. Fernández-Sánchez, V. S. Silva, M. Cicuéndez, M. Vila, A. Martínez-Alonso and J. M. D. Tascón, Aqueous Exfoliation of Transition Metal Dichalcogenides Assisted by DNA/RNA Nucleotides: Catalytically Active and Biocompatible Nanosheets Stabilized by Acid−Base Interactions, ACS Appl. Mater. Interfaces, 2017, 9, 2835–2845.
[23] S. García-Dalí, J. I. Paredes, J. M. Munuera, Silvia Villar-Rodil, A. Adawy, A. Martínez-Alonso, J. M.D. Tascón. Aqueous cathodic exfoliation strategy toward solution-processable and phase-preserved MoS_2 nanosheets for energy storage and catalytic applications. ACS Appl. Mater. Interfaces 2019, 11, 40, 36991–37003.
[24] J. Liu, X. Yan, L. Wang, L. Kong and P. Jian, Three-dimensional nitrogen-doped graphene foam as metal-free catalyst for the hydrogenation reduction of *p*-nitrophenol, J. Colloid Interface Sci., 2017, 497, 102–107.
[25] J. Song, S.W. Kang, Y.W. Lee, Y. Park, J.-H. Kim and S.W. Han, Regulating the Catalytic Function of Reduced Graphene Oxides Using Capping Agents for Metal-Free Catalysis, ACS Appl. Mater. Interfaces, 2017, 9, 1692–1701.
[26] K. Peng, L. Fu, H. Yang, J. Ouyang and A. Tang, Hierarchical MoS2 intercalated clay hybrid nanosheets with enhanced catalytic activity, Nano Res., 2017, 10, 570–583.
[27] C. Nethravathi, A.D. Manganahalli, M. Rajamathi, Bi_2Te_3-MoS_2 layered nanoscale heterostructures for electron transfer catalysis, ACS Appl. Nano Mater., 2019, 2, 2005–2012.

[28] N. Sahiner, H. Ozay, O. Ozay, N. Aktas, A soft hydrogel reactor for cobalt nanoparticle preparation and use in the reduction of nitrophenols, Appl. Catal. B-Environ. 101 (2010) 137–143.
[29] S. Zhang, S. Gai, F. He, S. Ding, L. Lia, P. Yang, In situ assembly of well-dispersed Ni nanoparticles on silica nanotubes and excellent catalytic activity in 4-nitrophenol reduction, Nanoscale, 2014, 6, 11181-11188.
[30] D. B. Jiang, X. Liu, Y. Yuan, L. Feng, J. Ji, J. Wang, D. Losic, H.-C. Yao, Y. X. Zhang, Biotemplated top-down assembly of hybrid Ni nanoparticles/N doping carbon on diatomite for enhanced catalytic reduction of 4-nitrophenol, Chem. Eng. J. 383 (2020) 123156.
[31] X. Cheng, A. Fu, H. Li, Y. Wang, P. Guo, J. Liu, J. Zhang, X. S, Zhao, Sustainable Preparation of Copper Particles Decorated Carbon Microspheres and Studies on Their Bactericidal Activity and Catalytic Properties ACS Sustainable Chem. Eng. 2015, 3, 2414−2422.
[32] B. K. Barman, K. K. Nanda, Uninterrupted galvanic reaction for scalable and rapid synthesis of metallic and bimetallic sponges/dendrites as efficient catalysts for 4-nitrophenol reduction, Dalton Trans., 2015, 44, 4215–4222.
[33] X. Cheng, A. Fu, H. Li, Y. Wang, P. Guo, J. Liu, J. Zhang, X. S. Zhao, Sustainable Preparation of Copper Particles Decorated Carbon Microspheres and Studies on Their Bactericidal Activity and Catalytic Properties, ACS Sustainable Chem. Eng. 2015, 3, 2414–2422.
[34] M. Guo, Y. Zhao, F. Zhang, L. Xu, H. Yang, X. Song, Y. Bu, Reduced graphene oxide-stabilized copper nanocrystals with enhanced catalytic activity and SERS properties, RSC Adv., 2016, 6, 50587–50594.
[35] J. Xia, G. He, L. Zhang , X. Sun, X. Wang, Hydrogenation of nitrophenols catalyzed by carbon black-supported nickel nanoparticles under mild conditions, Appl. Catal. B-Environ. 180 (2016) 408–415.
[36] S. Bae , S. Gim , H. Kim , K. Hanna. Effect of $NaBH_4$ on properties of nanoscale zero-valent iron and its catalytic activity for reduction of p-nitrophenol. Appl. Catal. B-Environ. 182 (2016) 541–549.

[37] H. Fang, M. Wen, Hanxing Chen, Qingsheng Wu and Weiying Li, Graphene stabilized ultra-small CuNi nanocomposite with high activity and recyclability toward catalysing the reduction of aromatic nitro-compounds, Nanoscale. 2016, 8, 536–542.

[38] W. Zuo, G. Yu and Z. Dong A MOF-derived nickel based N-doped mesoporous carbon catalyst with high catalytic activity for the reduction of nitroarenes, RSC Adv., 2016, 6, 11749–11753.

[39] X. Li, C. Zeng, J. Jiang, L. Ai, Magnetic cobalt nanoparticles embedded in hierarchically porous nitrogen-doped carbon frameworks for highly efficient and well-recyclable catalysis, J. Mater. Chem. A, 2016, 4, 7476–7482.

[40] Z. Ji, Y. Wang, X. Shen, H. Ma, J. Yang, A. Yuan, H. Zhou, Facile synthesis and enhanced catalytic performance of reduced graphene oxide decorated with hexagonal structure Ni nanoparticles, J. Colloid Interf. Science 487 (2017) 223–230.

[41] J. Jiang, Y. S. Lim, S. Park, S.-H. Kim, S. Yoon, L. Piao. Hollow porous Cu particles from silica-encapsulated Cu_2O nanoparticle aggregates effectively catalyze 4-nitrophenol reduction, Nanoscale, 2017, 9, 3873–3880.

[42] M. Du, Q. Liu, C. Huang, X. Qiu, One-step synthesis of magnetically recyclable Co@BN core–shell nanocatalysts for catalytic reduction of nitroarenes, RSC Adv., 2017, 7, 35451–35459.

[43] J. Liu, Z. Wang, X. Yan, P. Jian, Metallic cobalt nanoparticles imbedded into ordered mesoporous carbon: A non-precious metal catalyst with excellent hydrogenation performance, J. Colloid Interf. Sci. 505 (2017) 789–795.

[44] G. Wu, X. Liang, L. Zhang, Z. Tang, M. Al-Mamun, H. Zhao, X. Su, Fabrication of Highly Stable Metal Oxide Hollow Nanospheres and Their Catalytic Activity toward 4-Nitrophenol Reduction, ACS Appl. Mater. Interfaces 2017, 9, 18207–18214.

[45] P. C. Rath, D. Saikia, M. Mishra, H.-M. Kao, Exceptional catalytic performance of ultrafine Cu_2O nanoparticles confined in cubic mesoporous carbon for 4-nitrophenol reduction, Appl. Surf. Sci. 427 (2018) 1217–1226.

[46] X. Jiang, B. Han, C. Zhou, K. Xia, Q. Gao, J. Wu. Cu Nanoparticles Supported on Oxygen-Rich Boron Nitride for the Reduction of 4‑Nitrophenol. ACS Appl. Nano Mater. 2018, 1, 6692–6700.

[47] L. Liu, Q. Zhao, R. Liu, L. Zhu. Hydrogen adsorption-induced catalytic enhancement over Cu nanoparticles immobilized by layered Ti_3C_2 MXene, Appl. Catal. B-Environ. 252 (2019) 198–204.

[48] C. Chu, S. Rao, Z. Ma, H. Han, Copper and cobalt nanoparticles doped nitrogen-containing carbon frameworks derived from CuO-encapsulated ZIF-67 as high-efficiency catalyst for hydrogenation of 4-nitrophenol, Appl. Catal. B-Environ. 256 (2019) 117792.

[49] Q. Chen, Y. Li, Q. Li, Y. Jia, X. Qiao. 3D Hierarchical N, O Co–Doped MoS_2/NiO Hollow Microspheres as Reusable Catalyst for Nitrophenols Reduction. ChemistrySelect 2019, 4, 9339 –9347.

[50] A. A. Jeffery, S. R. Rao, M. Rajamathi, Preparation of MoS_2–reduced graphene oxide (rGO) hybrid paper for catalytic applications by simple exfoliation–costacking. Carbon 112 (2017) 8–16.

[51] H. Oudghiri-Hassani, F. Al Wadaani. Preparation, Characterization and Catalytic Activitynof Nickel Molybdate ($NiMoO_4$) Nanoparticles. Molecules 2018, 23, 273.

[52] A. Goyal, S. Bansal, S. Singhal, Facile reduction of nitrophenols: Comparative catalytic efficiency of MFe_2O_4 (M [Ni, Cu, Zn) nanoferrites. International Journal of Hydrogen Energy 39 (2014) 4895-4908.

[53] S. U. Nandanwar, M. Chakraborty, Synthesis of Colloidal CuO/γ-Al_2O_3 by Microemulsion and Its Catalytic Reduction of Aromatic Nitro Compounds. Chin. J. Catal., 2012, 33, 1532–1541.

[54] J. Xia , G. He , L. Zhang , X. Sun, X. Wang, Hydrogenation of nitrophenols catalyzed by carbon black-supported nickel nanoparticles under mild conditions. Appl. Catal. B 180 (2016) 408–415.

[55] H. Oudghiri-Hassani. Synthesis, characterization and catalytic performance of iron molybdate $Fe_2(MoO_4)_3$ nanoparticles. Catal. Commun. 60 (2015) 19–22.

[56] M. Karthik, P. Suresh. Greener Synthesis of Reduced Graphene Oxide-Nickel Nanocomposite: Rapid and Sustainable Catalyst for the Reduction of Nitroaromatics. ChemistrySelect 2017, 2, 6916 – 6928.
[57] J. Jiao, H. Wang, W. Guo, R. Li, K. Tian, Z. Xu, Y. Jia, Y. Wu, L. Cao. In Situ Confined Growth Based on a Self-Templating Reduction Strategy of Highly Dispersed Ni Nanoparticles in Hierarchical Yolk–Shell Fe@SiO_2 Structures as Efficient Catalysts. Chem. Asian J. 2016, 11, 3534 – 3540.
[58] M. Ajmal, M. Siddiq, H. Al-Lohedan and N. Sahiner. Highly versatile p(MAc)–M (M: Cu, Co, Ni) microgel composite catalyst for individual and simultaneous catalytic reduction of nitro compounds and dyes. RSC Adv., 2014, 4, 59562–59570
[59] Ö. Karahan, E. Biçer, A. Taşdemir, A. Yürüm, S. A. Gürsel. Development of Efficient Copper-Based MOF-Derived Catalysts for the Reduction of Aromatic Nitro Compounds. Eur. J. Inorg. Chem. 2018, 1073–1079.
[60] P. Das, S. Ghosh, M. Baskey (Sen). Heterogeneous catalytic reduction of 4-nitroaniline by RGO-Ni nanocomposite for water resource management. Journal of Materials Science: Materials in Electronics (2019) 30:19731–19737.
[61] K. Mishra, T. N. Poudel, N. Basavegowda, Y. R. Lee. Enhanced catalytic performance of magnetic Fe_3O_4–MnO_2 nanocomposites for the decolorization of rhodamine B, reduction of 4-nitroaniline, and sp C–H functionalization of 2-methylpyridines to isatins. Journal of Catalysis 344 (2016) 273–285.
[62] M. Tumma, R. Srivastava. Transition metal nanoparticles supported on mesoporous polyaniline catalyzed reduction of nitroaromatics. Catalysis Communications 37 (2013) 64–68.
[63] S. Venkatakrishnan, G. Veerappan, E. Elamparuthi, A. Veerappan. Aerobic synthesis of biocompatible copper nanoparticles: promising antibacterial agent and catalyst for nitroaromatic reduction and C–N cross coupling reaction. RSC Adv., 2014, 4, 15003–15006.
[64] U. Kurtan, E. Onus¸ Md. Amir, A. Baykal. Fe_3O_4@Hpipe-4@Cu Nanocatalyst for Hydrogenation of Nitro-Aromatics and Azo Dyes. J Inorg Organomet Polym, 2015, 25, 1120–1128.

[65] P. Zarringhadam, S. Farhadi. Hydrothermal Synthesis of Novel Magnetic Plate-Like $Bi_2O_2CO_3/CoFe_2O_4$ Hybrid Nanostructures and their Catalytic Performance for the Reduction of Some Aromatic Nitrocompounds. Acta Chim. Slov. 2018, 65, 448–461.
[66] M. Niakan, Z. Asadi. Selective Reduction of Nitroarenes Catalyzed by Sustainable and Reusable DNA-supported Nickel Nanoparticles in Water at Room Temperature. Catalysis Letters (2019) 149:2234–2246.
[67] R. J. Kalbasi, A. A. Nourbakhsh, F. Babaknezhad, Synthesis and characterization of Ni nanoparticles-polyvinylamine/SBA-15 catalystbfor simple reduction of aromatic nitro compounds. Catalysis Communications 12 (2011) 955–960.
[68] F. Zamani, S. Kianpou. Fast and efficient reduction of nitro aromatic compounds over Fe_3O_4/β-alanine-acrylamide-Ni nanocomposite as a new magnetic catalyst. Catalysis Communications 45 (2014) 1–6.
[69] K. Peng, L. Fu, H. Yang, J. Ouyang, A. Tang. Hierarchical MoS_2 intercalated clay hybrid nanosheets with enhanced catalytic activity. Nano Res., 2017, 10, 570–583.
[70] E. Kalantari , M. A. Khalilzadeh , D. Zareyee , M. Shokouhimehr, Catalytic degradation of organic dyes using green synthesized Fe_3O_4-cellulose-copper nanocomposites, Journal of Molecular Structure 1218 (2020) 128488.
[71] H. Wang, N. Wang, F. Wang, F. Xiao, D. Pan. Spherical montmorillonite-supported molybdenum disulfide nanosheets as a self-sedimentary catalyst for organic pollutants removal. Separation and Purification Technology 251 (2020) 117346.
[72] N. Saha, A. Sarkar, A. B. Ghosh, P. Mondal, J. Satra, B. Adhikary Advanced catalytic performance of amorphous MoS_2 for degradation/reduction of organic pollutants in both individual and simultaneous fashion. Ecotoxicology and Environmental Safety 160 (2018) 290–300.
[73] V. Balakumar, J. W. Ryu, H. Kim, R. Manivannan, Y.-A. Son Ultrasonic synthesis of α-MnO_2 nanorods: An efficient catalytic conversion of refractory pollutant, methylene blue. Ultrasonics Sonochemistry 62 (2020) 104870.
[74] X. Yang, H. Zhong, Y. Zhu, H. Jiang, J. Shen, J. Huang, C. Li. Highly efficient reusable catalyst based on silicon nanowire arrays decorated with copper nanoparticles. J. Mater. Chem. A, 2014, 2, 9040–9047.

[75] M. Makarova, Y. Okawa, M. Aono , Selective adsorption of thiol molecules at sulfur vacancies on MoS_2 (0001), followed by vacancy repair via S-C dissociation, J. Phys. Chem. C 116 (2012) 22411–22416.

[76] Z. Yu, Y. Pan, Y. Shen, Z. Wang, Z.-Y. Ong, T. Xu, R. Xin, L. Pan, B. Wang, L. Sun, J. Wang, G. Zhang, Y.W. Zhang, Y. Shi, X. Wang. Towards intrinsic charge transport in monolayer molybdenum disulfide by defect and interface engineering, Nat. Commun. 5 (2014) 5290.

[77] K. Cho, M. Min, T.-Y. Kim, H. Jeong, J. Pak, J.-K. Kim, J. Jang, S.J. Yun, Y.H. Lee, W.-K. Hong, T. Lee, Electrical and optical characterization of MoS_2 with sulfur vacancy passivation by treatment with alkanethiol molecules, ACS Nano 9 (2015) 8044–8053.

[78] P. Hervés, M. Pérez-Lorenzo, L. M. Liz-Marzán, L., J. Dzubiella, Y. Lubc, M. Ballauff. Catalysis by Metallic Nanoparticles in Aqueous Solution: Model Reactions. Chem. Soc. Rev. 2012, 41, 5577−5587.

[79] S. Carregal-Romero, N. J. Buurma, J. Pérez-Juste, L. M. Liz-Marzán, P. Hervés. Catalysis by Au@pNIPAM Nanocomposites: Effect of the Cross-Linking Density. Chem. Mater. 2010, 22, 3051−3059.

[80] J.I. Paredes, J.M. Munuera, S. Villar-Rodil, L. Guardia, M. Ayán-Varela, A. Pagán, S.D. Aznar-Cervantes, J.L. Cenis, A. Martínez-Alonso, J.M.D. Tascón, Impact of covalent functionalization on the aqueous processability, catalytic activity, and biocompatibility of chemically exfoliated MoS_2 nanosheets, ACS Appl. Mater. Interfaces 8 (2016), 27974-27986.

[81] M. Chatenet, F. Micoud, I. Roche, E. Chainet. Kinetics of Sodium Borohydride Direct Oxidation and Oxygen Reduction in Sodium Hydroxide Electrolyte: Part I. BH_4^- Electro-Oxidation on Au and Ag Catalysts. Electrochim. Acta 2006, 51, 5459−5467.

[82] J. A. Gardiner, J. W. Collat. The Hydrolysis of Sodium Tetrahydroborate. Identification of an Intermediate. J. Am. Chem. Soc. 1964, 86, 3165−3166.

[83] S.W. Han, G.-B. Cha, Y. Park, S.C. Hong, Hydrogen physisorption based on the dissociative hydrogen chemisorption at the sulphur vacancy of MoS_2 surface, Sci. Rep. 7 (2017) 7152 .

[84] N.P. Rezende, A.R. Cadore, A.C. Gadelha, C.L. Pereira, V. Ornelas, K. Watanabe, T. Taniguchi, A.S. Ferlauto, A. Malachias, L.G. Campos, R.G. Lacerda. Probing the electronic properties of monolayer MoS 2 via interaction with molecular hydro- gen, Adv. Electron. Mater. 5 (2019) 1800591.
[85] C. Zhu, D. Gao, J. Ding, D. Chao, J. Wang, TMD-based highly efficient electro-catalysts developed by combined computational and experimental approaches, Chem. Soc. Rev. 47 (2018) 4332–4356.
[86] S. Jayabal, G. Saranya, J. Wu, Y. Liu, D. Geng, X. Meng, Understanding the high--electrocatalytic performance of two-dimensional MoS_2 nanosheets and their composite materials, J. Mater. Chem. A 5 (2017) 24540–24563.
[87] D.A. Finkelstein, N. Da Mota, J.L. Cohen, H.D. Abruña, Rotating disk electrode (RDE) investigation of BH_4^- and BH_3OH^- electro-oxidation at Pt and Au: implications for BH_4^- fuel cells, J. Phys. Chem. C 113 (2009) 19700–19712.
[88] Y. Fu, T. Huang, B. Jia, J. Zhu, X. Wang, Reduction of nitrophenols to aminophenols under concerted catalysis by Au/g-C_3N_4 contact system, Appl. Catal. B: Environ. 202 (2017) 430–437.
[89] Y. Cai, Z. Bai, H. Pan, Y.P. Feng, B.I. Yakobson, Y.-W. Zhang, Constructing metal- lic nanoroads on a MoS_2 monolayer via hydrogenation, Nanoscale 6 (2014) 1691–1697.
[90] T. Aditya, A. Pal, T. Pal, Nitroarene reduction: a trusted model reaction to test nanoparticle catalysts, Chem. Commun. 51 (2015) 9410–9431.
[91] J. Hong, Z. Hu, M. Probert, K. Li, D. Lv, X. Yang, L. Gu, N. Mao, Q. Feng, L. Xie, J. Zhang, D. Wu, Z. Zhang, C. Jin, W. Ji, X. Zhang, J. Yuan, Z. Zhang, Exploring atomic defects in molybdenum disulphide monolayers, Nat. Commun. 6 (2015), 6293.
